# Camrelizumab plus gemcitabine and oxaliplatin (GEMOX) in patients with advanced biliary tract cancer: a single-arm, open-label, phase II trial

**DOI:** 10.1136/jitc-2020-001240

**Published:** 2020-11-10

**Authors:** Xiaofeng Chen, Xiaofeng Wu, Hao Wu, Yanhong Gu, Yang Shao, Qianwen Shao, Feipeng Zhu, Xiao Li, Xiaofeng Qian, Jun Hu, Fengjiao Zhao, Weidong Mao, Jing Sun, Jian Wang, Gaohua Han, Changxian Li, Yongxiang Xia, Poshita Kumari Seesaha, Dongqin Zhu, Huajun Li, Junling Zhang, Guoqiang Wang, Xuehao Wang, Xiangcheng Li, Yongqian Shu

**Affiliations:** 1 Department of Oncology, The First Affiliated Hospital of Nanjing Medical University, Nanjing, China; 2 Department of Oncology, Pukou Branch Hospital of Jiangsu Province Hospital, Nanjing, China; 3 Collaborative Innovation Center for Cancer Personalized Medicine, Nanjing Medical University, Nanjing, China; 4 Hepatobiliary Center, The First Affiliated Hospital of Nanjing Medical University, Nanjing, China; 5 Medical Department, Nanjing Geneseeq Technology Inc, Nanjing, China; 6 Department of Radiology, The First Affiliated Hospital of Nanjing Medical University, Nanjing, China; 7 Department of Pathology, The First Affiliated Hospital of Nanjing Medical University, Nanjing, China; 8 Department of Oncology, Nanjing Red Cross Hospital, Nanjing, China; 9 Department of Oncology, Jiangyin People’s Hospital, Jiangyin, China; 10 Department of Oncology, Taizhou People's Hospital Affiliated to Nantong University, Taizhou, China; 11 Medical Department, Jiangsu Hengrui Medicine Co Ltd, Lianyungang, China; 12 Medical Department, 3D Medicines Inc, Shanghai, China; 13 Medical Department, Burning Rock Biotech, Guangzhou, China

**Keywords:** biomarkers, tumor, clinical trials, phase II as topic, immunotherapy

## Abstract

**Background:**

Immune checkpoint inhibitors monotherapy has been studied in patients with advanced biliary tract cancer (BTC). The aim of this study was to assess the efficacy and safety of camrelizumab, plus gemcitabine and oxaliplatin (GEMOX) as first-line treatment in advanced BTC and explored the potential biomarkers associated with response.

**Methods:**

In this single-arm, open-label, phase II study, we enrolled stage IV BTC patients. Participants received camrelizumab (3 mg/kg) plus gemcitabine (800 mg/m^2^) and oxaliplatin (85 mg/m^2^). Primary endpoints were 6-month progression-free survival (PFS) rate and safety. Secondary endpoints were objective response rate (ORR), PFS and overall survival (OS). Exploratory endpoints included association between response and tumor mutational burden (TMB), blood TMB, dynamic change of ctDNA and immune microenvironment.

**Results:**

54 patients with advanced BTC were screened, of whom 38 eligible patients were enrolled. One patient withdrew informed consent before first dose treatment. Median follow-up was 11.8 months. The 6-month PFS rate was 50% (95% CI 33 to 65). Twenty (54%) out of 37 patients had an objective response. The median PFS was 6.1 months and median OS was 11.8 months. The most common treatment-related adverse events (TRAEs) were fatigue (27 (73%)) and fever (27 (73%)). The most frequent grade 3 or worse TRAEs were hypokalemia (7 (19%)) and fatigue (6 (16%)). The ORR was 80% in patients with programmed cell death ligand-1 (PD-L1) tumor proportion score (TPS) ≥1% versus 53.8% in PD-L1 TPS <1%. There was no association between response and TMB, blood TMB, immune proportion score or immune cells (p>0.05), except that PFS was associated with blood TMB. Patients with positive post-treatment ctDNA had shorter PFS (p=0.007; HR, 2.83; 95% CI 1.27 to 6.28).

**Conclusion:**

Camrelizumab plus GEMOX showed a promising antitumor activity and acceptable safety profile as first-line treatment in advanced BTC patients. Potential biomarkers are needed to identify patients who might respond to camrelizumab plus GEMOX.

**Trial registration number:**

NCT03486678.

## Introduction

Biliary tract cancer (BTC) is a rare type of malignant tumor accounting for about 3% of all gastrointestinal malignancies.^1^ The global incidence rate was 21.1% and the mortality rate was 17.4% in 2017.^2^ Due to the aggressive nature of BTC, most patients present with an advanced BTC and cannot be surgically resected when diagnosed.^3^ For these patients, gemcitabine plus cisplatin or gemcitabine plus oxaliplatin (GEMOX) have shown prolonged median overall survival (mOS, 11.7 months and 9.5 months, respectively) and have become the first-line options for advanced BTC patients.^4 5^ However, the toxicity of gemcitabine plus cisplatin are higher than GEMOX.^6^ In practice, 6–8 cycles of GEMOX regimen are more frequently selected in the treatment of patients with BTC with an acceptable safety profile.^7^ However, the OS is far from satisfactory and the 5-year survival rate is less than 10%.^3^ Hence, there remains an unmet need to develop more effective treatment options for patients with unresectable BTC.

Immune checkpoint inhibitors (ICIs) such as programmed cell death-1 (PD-1) and programmed cell death ligand-1 (PD-L1) antibodies, have gained great success in various types of cancers.^8^ In patients with BTC, pembrolizumab has been approved in patients with micro-satellite instability high or deficiency in mismatch repair according to pan-cancer studies.^9^ Several clinical studies involving ICI as second-line treatment in patients with BTC also show promising results, like for instance the KEYNOTE 028 and KEYNOTE 158 trials with pembrolizumab and a phase II trial with nivolumab.^10–12^ Therefore, the application of ICIs in BTC looks promising.

Recently, immunotherapy combined with chemotherapy has been investigated in many types of cancers and has shown promising antitumor efficacy.^13 14^ For example, in the KEYNOTE0189 study, pembrolizumab combined with pemetrexed plus carboplatin, as first-line therapy, prolonged the OS in non-small cell lung cancer (NSCLC).^15^ SHR-1210 (camrelizumab) combined with gemcitabine plus cisplatin yielded promising safety and efficacy in recurrent or metastatic nasopharyngeal carcinoma.^14^ However, whether the combination of ICIs with chemotherapy would improve the clinical outcomes in advanced BTC has not yet been explored.

Herein we report the results from a prospective single-arm open label trial assessing the safety and antitumor activity of camrelizumab, a fully humanized IgG4-κ PD-1 monoclonal antibody with high affinity combined with GEMOX regimen in advanced BTC patients.

## Methods

### Study design and participants

This study was a single-arm, phase II trial assessing the safety and antitumor activity of camrelizumab plus GEMOX in patients with advanced BTC(online supplemental file 2). Eligible patients were aged 18–75 years with histologically confirmed stage IV advanced BTC including cholangiocarcinoma and gallbladder cancer. Additional inclusion criteria included an Eastern Cooperative Oncology Group performance status of 0 or 1 and presence of at least one measurable lesion assessed using the Response Evaluation Criteria in Solid Tumors version 1.1 (RECIST version 1.1). Patients were required to have an estimated life expectancy of 12 weeks or more and adequate organ function (neutrophil count of ≥1.5×10^9^ cells/L, hemoglobin concentrations of ≥90 g/L, platelet cell count of ≥100×10^9^ cells/L, bilirubin <1.5×ULN, blood glutamate transaminase <2.5×ULN (could be extended to 5×ULN in case of liver metastases), creatinine clearance rate >60 mL/min (calculated using Cockcroft-Gault formula) and left ventricular ejection fraction ≥50%). Patients who received previous treatment with drugs specifically targeting T-cell costimulation or checkpoint pathways and those with previous or concurrent malignancies (except curatively treated skin basal cell carcinoma or cervical carcinoma in situ) were excluded from the study.

10.1136/jitc-2020-001240.supp2 Supplementary data

### Procedures

Enrolled participants received camrelizumab (3 mg/kg, total dose ≤200 mg, Intravenous drips (ivd), D1/2W) plus gemcitabine (800 mg/m^2^, ivd, D1/2W) and oxaliplatin (85 mg/m^2^, ivd, D2/2W). Chemotherapy lasted for no more than 12 cycles. Once chemotherapy intolerance occurred or at end of 12 cycles, patients with objective response or stable disease would continue to receive camrelizumab (3 mg/kg, total dose ≤200 mg) as single agent until confirmed disease progression, death, unacceptable toxicity, withdrawal of consent, investigator’s decision or completion of 24 months of study. We assessed response to treatment every 8 weeks using CT or MRI based on RECIST (version 1.1) criteria.

Adverse events were monitored and graded using National Cancer Institute Common Terminology Criteria for Adverse Events (version 4.0). All adverse events, from the time of treatment allocation till 90 days after cessation of treatment, were reported.

### Outcomes

The primary endpoints were 6-month progression-free survival (PFS) rate, determined by RECIST version 1.1 and safety. Secondary end points were objective response rate (ORR), PFS, OS and duration of response. PFS was defined as the time from first drug administration to the first documented disease progression according to RECIST version 1.1 or to death from any cause, whichever occurred first. OS was defined as time from first drug administration to death from any cause. Duration of response was defined as time from first RECIST response to progression in patients who achieved a partial response or better. Exploratory endpoints included the association between survival and PD-L1 expression, tumor mutational burden (TMB), blood tumor mutational burden (bTMB), change of ctDNA and immune microenvironment.

Optional baseline biopsy specimens and blood samples were obtained from patients for exploratory biomarker assessment. Specifically, genomic DNA from tumor tissues were extracted and matched with white blood cell samples, followed by next generation sequencing and analysis of the TMB. Tissue PD-L1 expression, CD8^+^ cell, macrophages and natural killer cells level were measured by multiple immunofluorescence. The ctDNA sequencing from blood samples before and 8–10 weeks after treatment was also performed.

### Statistical analysis

A total of 35 patients would provide 80% power to detect a 6-month progression-free rate of 60% at a one-sided 2.5% alpha level under the null hypothesis of the 6-month progression-free rate equals to 40%. Considering a 10% discontinuation rate, 38 assessable patients were enrolled in the study. Activity was assessed in all patients who received at least one dose of camrelizumab, had measurable disease at baseline and had one or more post-baseline scans or discontinued because of progressive disease or a treatment-related adverse event (full analysis set). Safety was assessed in all patients who received at least one dose of camrelizumab (all-patients-as-treated population).

For objective response, 95% CIs were generated using the exact binomial distribution. Patients without response data were considered as non-responders. We used the Kaplan-Meier method for estimating PFS, OS and duration of response. Exploratory, post-hoc analysis of the association between clinical response and PD-L1 expression, TMB, bTMB or tumor immune microenvironment was done in the full analysis set. For all analyzes, p value <0.05 was considered to be statistically significant, and a CI of 95% was used (95% CI). The SPSS V.22.0 software was used for statistical analysis.

## Results

### Patients

**Figure 1:**
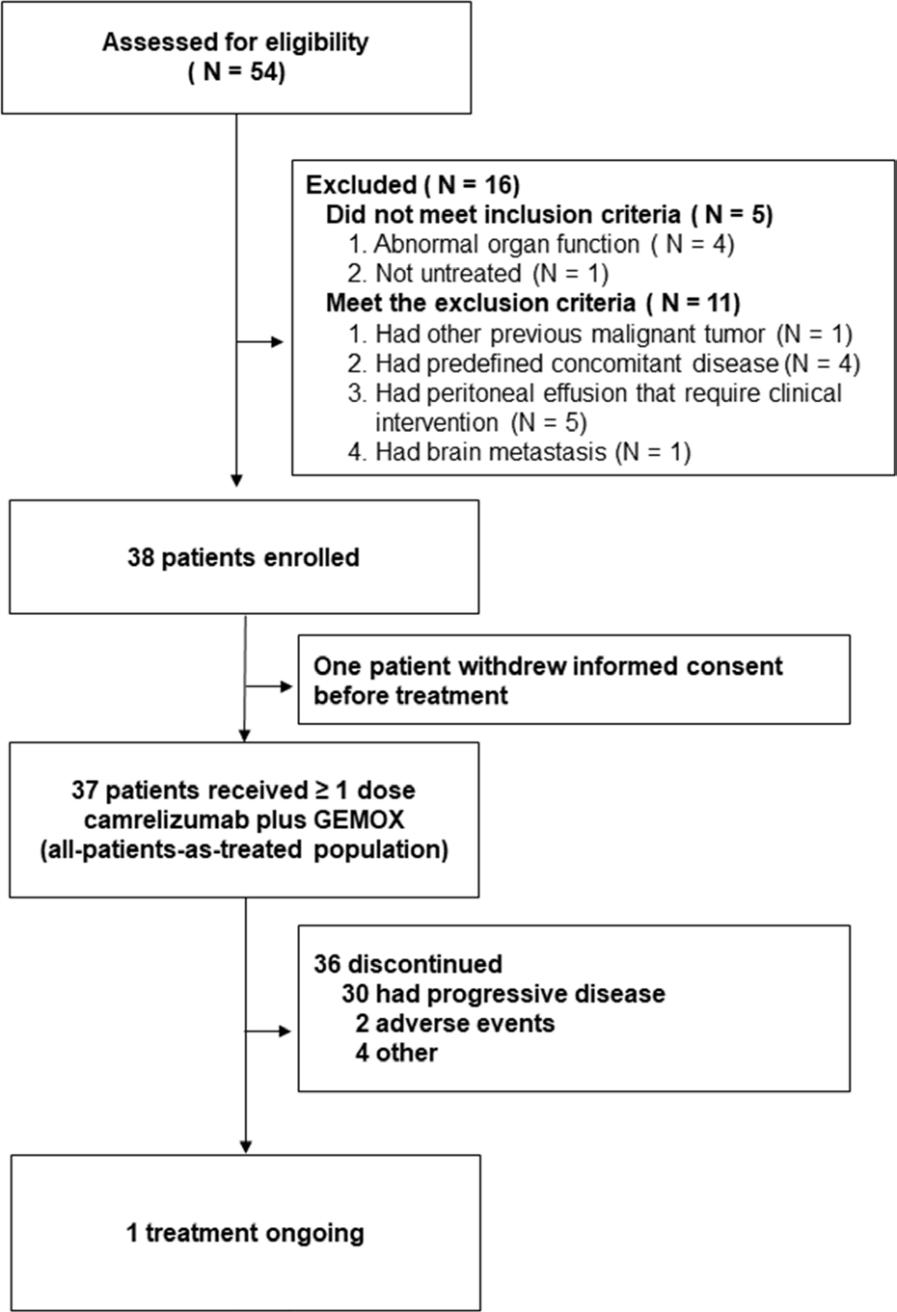
Flow of participants in the study.

Between February 2018 and April 2019, 54 patients were screened for eligibility. Of these patients, 16 (30%) were deemed ineligible because they did not meet the inclusion criteria or met the exclusion criteria (figure 1). One patient withdrew his consent before first treatment. Thirty-seven enrolled patients were treated with at least one dose of camrelizumab and were included in the primary analysis. The baseline characteristics of the enrolled participants are shown in online supplemental table S1.

10.1136/jitc-2020-001240.supp1 Supplementary data

As of data cut-off on August 1, 2020, the median duration of follow-up was 11.8 months (IQR 7.4–19.1) and on this date, 1 (2.7%) out of the 37 participants who had received at least one dose of camrelizumab were still receiving the treatment. The most common reasons for treatment discontinuation were progressive disease in 30 (81%) participants and adverse events in 2 (5%) participants (figure 1).

### Efficacy

**Figure 2:**
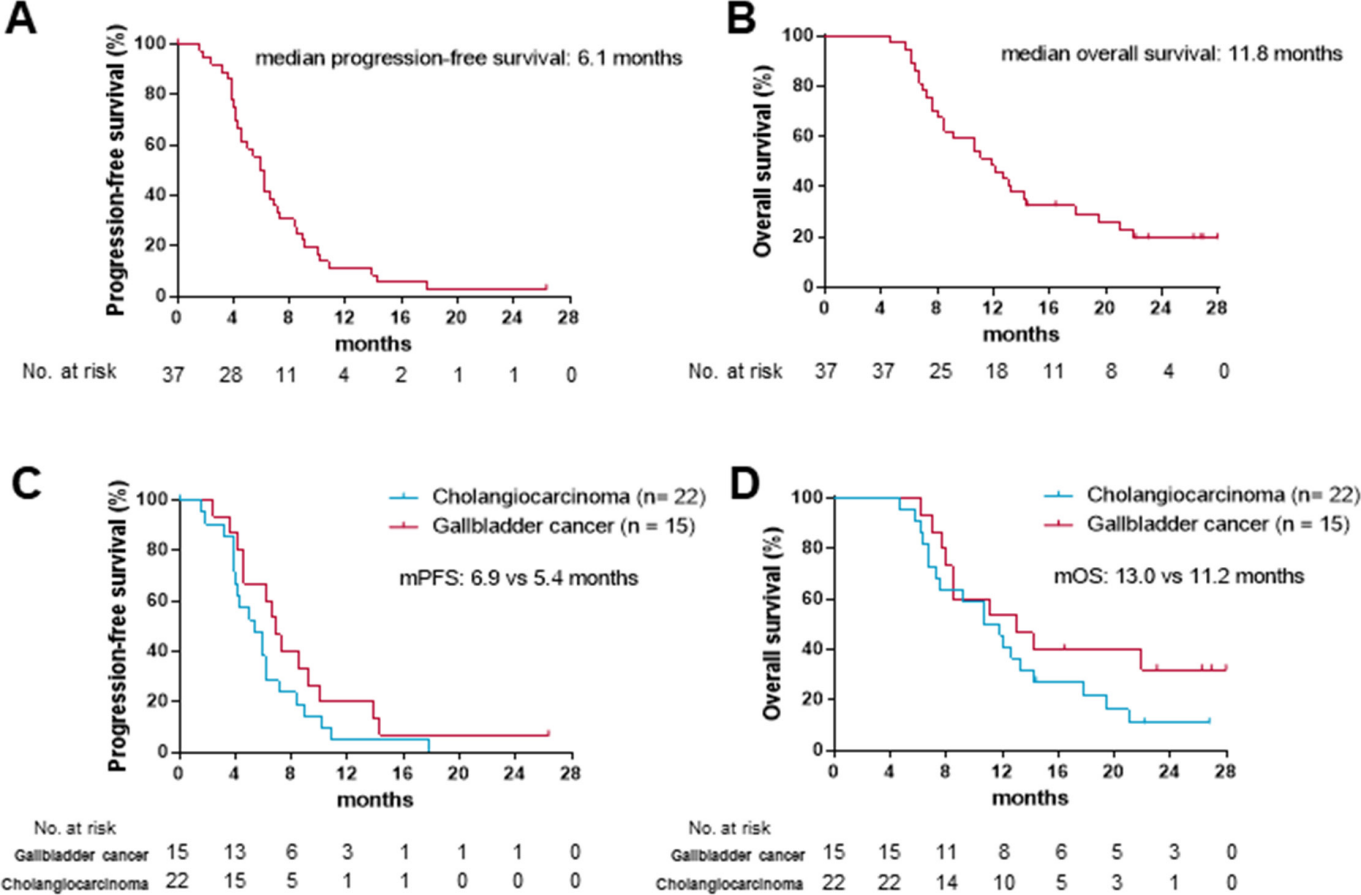
Kaplan-Meier curves for progression-free survival and overall survival in the intent-to-treat (ITT) population (A, B) and primary site subgroups (C, D).

As data cut-off, 30 (81%) out of 37 participants had died or had disease progression. At 6 months, 18 out of the 37 patients were still alive and progression-free, giving a 6-month PFS of 50% (95% CI 33 to 65). The median progression-free survival (mPFS) was 6.1 months (95% CI 5.1 to 6.8; figure 2A). As data cut-off, 29 (78%) out of the 37 participants in the study had died and the mOS was 11.8 months (95% CI 8.3 to 15.4; figure 2B). The mPFS was 6.9 months in patients with gallbladder cancer versus 5.4 months in patients with cholangiocarcinoma (figure 2C). The mOS was 13.0 months in gallbladder cancer versus 11.2 months in cholangiocarcinoma (figure 2D).

**Figure 3:**
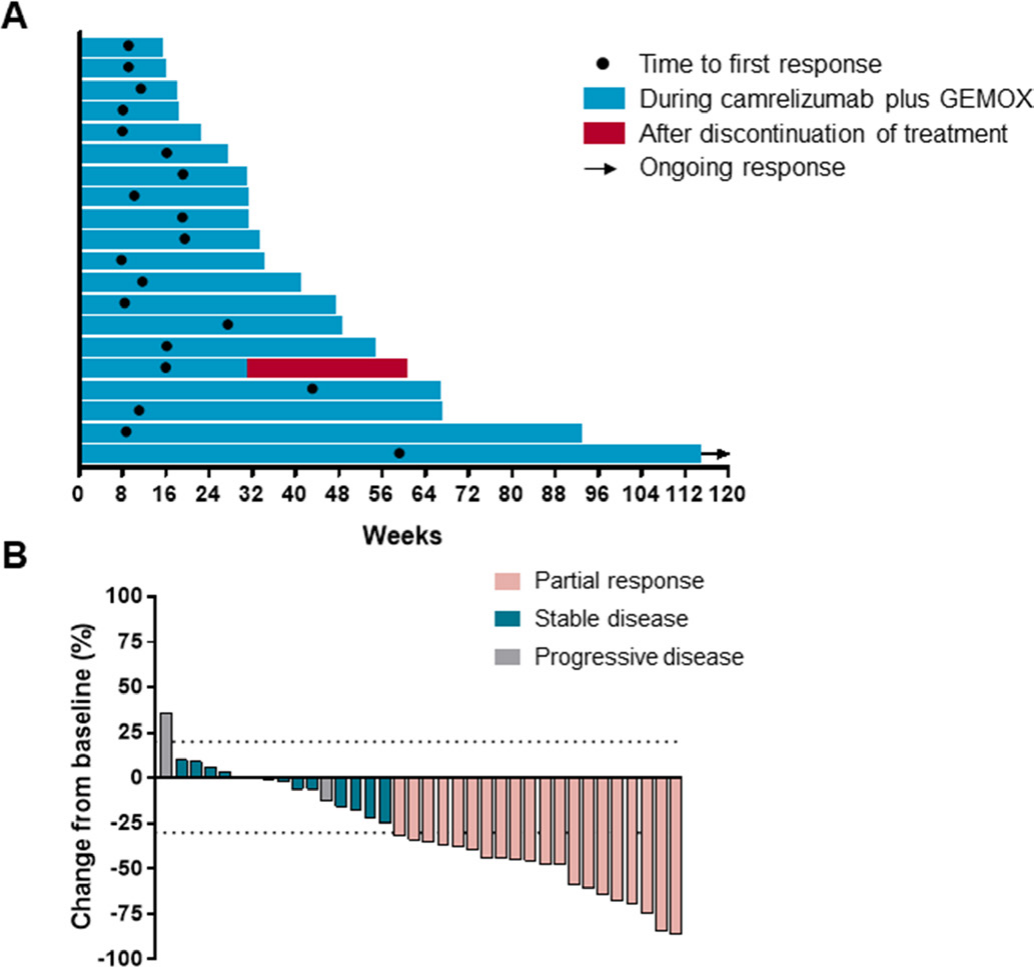
Characteristics of objective response in patients with camrelizumab plus gemcitabine and oxaliplatin (GEMOX). (A) Duration of response (N=20). (B) The maximum percentage reduction from baseline in target lesions (N=36). One patient discontinued because of treatment-related adverse effect before first radiographic assessment and was not included.

An objective response was recorded in 20 (54%) out of 37 participants (95% CI 38 to 69; table 1) who had received at least one dose of camrelizumab plus GEMOX. 20 (54%) participants had partial response, 13 (35%) had stable disease and 3 (8%) had progressive disease. One patient discontinued treatment because of treatment-related adverse event before first radiographic assessment. Disease control was reported in 33 (89%; 95% CI 75 to 96) out of the 37 treated participants (table 1). The median time to response was 2.7 months (range 1.8–13.7; figure 3A). As data cut-off, 1 of the 20 responses were ongoing and the median duration of response was 4.8 months (95% CI 1.4 to 19.5 months). 30 (83%) out of 36 assessable patients had a decrease in tumor size as compared with baseline and the median change from baseline was −35% (figure 3B).

**Table 1:** Clinical activity of camrelizumab plus gemcitabine and oxaliplatin (GEMOX) in patients with advanced biliary tract cancer

Variable	Camrelizumab+GEMOX (N=37)
Objective response	
No. of response	20
% of patients (95% CI)	54 (38 to 69)
Disease control	
No. of disease control	33
% of patients (95% CI)	89 (77 to 96)
Best overall response—no. (%)	
Complete response	0 (0)
Partial response	20 (54)
Stable disease	13 (35)
Progression disease	3 (8)
No assessment*	1 (3)
Time to response—months	
Median	2.7
Range	1.8–13.7
Duration of response—months	
Median	4.8
Range	1.4–19.5

*One patient discontinued before the first postbaseline radiographic assessment because of a treatment-related adverse event.

### Safety

At least one treatment-related adverse event occurred in 36 (97%) of 37 participants (grade 3–4 in 26 (70%) patients; table 2). The most common treatment-related adverse events were fatigue (27 (73%)) and fever (27 (73%)). The most frequent grade 3 or worse adverse events were hypokalemia (7 (19%)), fatigue (6 (16%)), nausea, neutropenia, gamma-glutamyl transferase (GGT) increased and biliary tract infection (5 (14%) each). Two (5%) participants discontinued treatment after an adverse event, including one (3%) patient with immune-related pneumonitis and one (3%) with skin reaction.

**Table 2:** Treatment-related adverse events

	All grade	Grade 3–4
Patients with ≥1 events	36 (97%)	26 (70%)
Fatigue	27 (73%)	6 (16%)
Fever	27 (73%)	2 (5%)
Thrombocytopenia	25 (68%)	4 (11%)
RCCEP	23 (62%)	0
Nausea	23 (62%)	5 (14%)
Hypocalcemia	23 (62%)	0
Amenia	22 (59%)	2 (5%)
Vomiting	20 (54%)	4 (11%)
Leukocytopenia	19 (51%)	4 (11%)
Hyponatremia	19 (51%)	1 (3%)
Neutropenia	18 (49%)	5 (14%)
AST increased	18 (49%)	1 (3%)
Hypoalbuminemia	16 (43%)	0
Constipation	14 (38%)	2 (5%)
ALT increased	14 (38%)	0
Neurotoxicity	12 (32%)	0
Hypokalemia	11 (30%)	7 (19%)
Alopecia	11 (30%)	0
Skin pigmentation	10 (27%)	0
Diarrhea	8 (22%)	1 (3%)
ALP increased	8 (22%)	0
GGT increased	8 (22%)	5 (14%)
Anorexia	7 (19%)	0
Peripheral neurotoxicity	6 (16%)	1 (3%)
Biliary tract infection	5 (14%)	5 (14%)
Blood bilirubin increased	5 (14%)	1 (3%)
Hypomagnesemia	5 (14%)	0
Insomnia	5 (14%)	0
Parodontopathy	5 (14%)	0
Hand-foot syndrome	4 (11%)	0
Hypophosphatemia	4 (11%)	2 (5%)
Mucositis	3 (8%)	1 (3%)
Rash	3 (8%)	2 (5%)

Data are n (%) in all treated patients (n=37). The table lists treatment-related adverse events reported in ≥10% patients or grade 3–4 treatment-related adverse events. Patients are counted for each applicable specific adverse event and could have more than one treatment-related event.

ALP, alkaline phosphatase; ALT, alanine aminotransferase; GGT, gamma-glutamyl transferase; RCCEP, reactive cutaneous capillary endothelial proliferation.

### Biomarkers

In the prespecified exploratory analysis, we evaluated the association between tumor immune microenvironment and clinical response to camrelizumab plus GEMOX in subsets of participants with tissue for immunohistochemistry. The proportion of patients who achieved an objective response was similar in the overall cohort (20 (54%) of 37 participants) and the subgroup with available PD-L1 expression data (18 (58%) of 31 participants), as were event rates for PFS (35 (95%) of 37 participants in the overall cohort; 29 (94%) of 31 participants in the PD-L1 subgroup). Association between biomarkers and clinical response along with survival are shown in online supplemental table S2. Objective responses were recorded in 80% (4 of 5 patients) with tumor proportion scores of at least 1% versus 55.8% (14 of 26 patients) with tumor proportion scores less than 1%, and 63% (5 of 8 patients) with immune proportion scores of at least 1% versus 57% (13 of 23 patients) with immune proportion scores less than 1%. The mPFS was 9.0 months in patients with tumor proportion scores of at least 1% versus 6.0 months with tumor proportion scores less than 1% (online supplemental table S2, figure S1A) and the mOS was 17.8 months versus 11.9 months (online supplemental table S2, figure S1B). The mPFS was 8.7 months in patients with immune proportion scores of at least 1% versus 6.0 months with immune proportion scores less than 1% (online supplemental table S2, figure S1C) and the mOS was 19.9 months versus 11.1 months (online supplemental table S2, figure S1D). No difference was observed in the number of cells and in the fraction of immune cells in center or invasive margin between responders and non-responders (p>0.05; online supplemental figure S2A, B).

**Figure 4:**
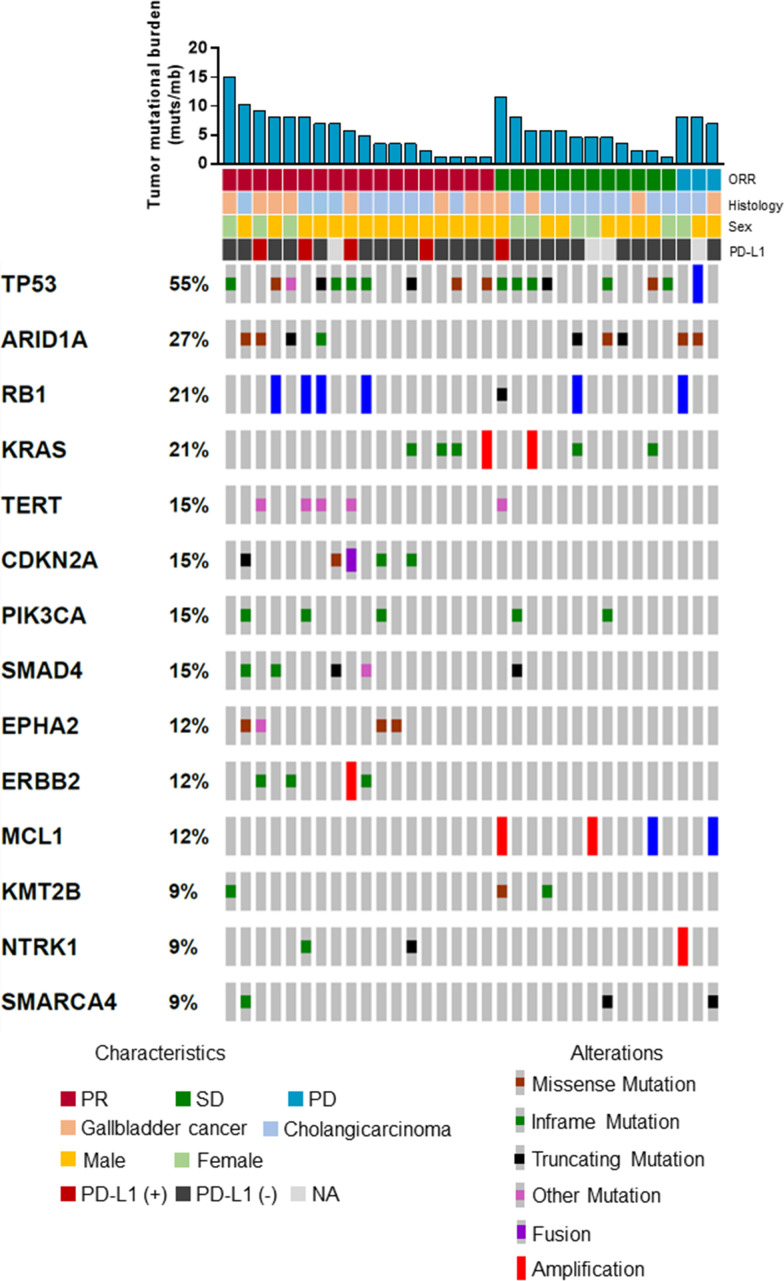
Distribution of genetic variations associated with treatment response. ORR, objective response rate; PD, progressive disease; PD-L1, programmed cell death ligand-1; PR, partial response; SD, stable disease.

In another prespecified exploratory analysis, we also evaluated the association between genomic alteration and clinical response to camrelizumab plus GEMOX in subsets of participants with baseline tissue for gene sequencing. The proportion of patients who achieved an objective response was similar for the overall cohort (20 (54%) of 37) and the subgroup with available gene sequencing data (18 (53%) of 34). Similar observations were obtained for PFS (35 (95%) of 37 participants in the overall cohort; 33 (97%) of 34 participants in the gene sequencing subgroup). Objective responses for participants were recorded in 60% (6 of 10) patients with top 25% TMB versus 50% (12 of 24) patients with bottom 75% TMB (online supplemental table S2). No association was observed between TMB and PFS or OS (PFS, HR, 0.89; 95% CI 0.41 to 1.92; p=0.76; online supplemental figure S3A); OS, HR, 0.90; 95% CI 0.39 to 3.05; p=0.79; online supplemental figure S3B). The median TMB was 5.3 muts/mb in responders versus 5.7 muts/mb in non-responders (p=0.92; online supplemental figure S3C). Distribution of genetic variations associated with the response to camrelizumab plus GEMOX is depicted in figure 4. The p values for the association between each gene alteration and PFS and OS are depicted in online supplemental figure S3D). Only *ARID1A* showed similar trend in the association with PFS and OS. The mPFS was 4.3 months in patients with *ARID1A* mutation versus 6.3 months in patients without *ARID1A* mutation (HR, 2.39; 95% CI 1.04 to 5.48; p=0.04; online supplemental figure S3E). The mOS was 7.2 months in patients with *ARID1A* mutation versus 13.2 months in patients without *ARID1A* mutation (HR, 2.60; 95% CI 1.11 to 6.10; p=0.03; online supplemental figure S3F).

We also evaluated the association between the dynamic change of ctDNA and clinical response to camrelizumab plus GEMOX in subsets of participants with post-treatment blood samples for ctDNA gene sequencing. The proportion of patients who achieved an objective response was also similar for the overall cohort (20 (54%) of 37) and the subgroup with available ctDNA data (17 (57%) of 30). Objective responses were observed in 10 out of 13 (77%) patients with negative post-treatment ctDNA versus 7 out of 17 (41%) patients with positive post-treatment ctDNA (online supplemental table S2). bTMB was correlated with tissue TMB (online supplemental figure S4A). There was no difference in bTMB between responders and non-responders (online supplemental figure S4B). The mPFS was 4.1 months in patients with top 25% bTMB versus 6.4 months in patients with bottom 75% bTMB (HR, 2.57; 95% CI 1.08 to 6.12; p=0.03; online supplemental figure S4C). No association between TMB and OS was observed (online supplemental figure S4D). However, the mPFS was 4.3 months in patients with positive post-treatment ctDNA versus 7.3 months in patients with negative post-treatment ctDNA (HR, 2.83; 95% CI 1.27 to 6.28; p=0.007; online supplemental figure S4E). The mOS was 9.1 months in patients with positive post-treatment ctDNA versus 13.0 months in patients with negative post-treatment ctDNA (HR, 1.77; 95% CI 0.78 to 3.99; p=0.16; online supplemental figure S4F). To further demonstrate the role of post-treatment ctDNA, we further studied the association between post-treatment ctDNA and PFS in responders and non-responders. In responders, patients with positive post-treatment ctDNA also had shorter PFS than patients with negative post-treatment ctDNA (5.0 versus 8.4 months, HR, 3.22; 95% CI 1.10 to 9.40; p=0.02; online supplemental figure S5A), while in non-responders, no difference was observed due to the small sample size (online supplemental figure S5B).

## Discussion

Camrelizumab plus GEMOX showed a promising antitumor activity with a manageable safety profile in patients with advanced BTC. Present treatment options for advanced BTC are limited. To our knowledge, this study is the first to report the efficacy and safety of an anti-PD-1 antibody plus chemotherapy in patients with advanced BTC.

The ORR of GEMOX chemotherapy regimen was 14.9%–18.9% in 2008, a phase II study reported that GEMOX regimen obtained an ORR of 14.9% (95% CI 7.4 to 25.7) in first-line treatment of advanced BTC,^16^ whereas the Korean cancer group yielded an ORR of 18.9%.^17^ The camrelizumab combination strategy dramatically improved the overall response rate by more than 30%, which remarkably expanded the proportion of those who benefited from this treatment regimen. Meanwhile, the mOS and mPFS were also prolonged by camrelizumab plus GEMOX compared with GEMOX alone ((12.1 months vs 8.8 months) and (6.0 months vs 3.4 months), respectively).^16^ The OS was extended by 3.3 months and the PFS was extended by 2.6 months, when compared with historical data.

Most trials used immunotherapy as second or beyond lines instead of first-line therapy. The ORR of pembrolizumab in the BTC cohort was 17% (95% CI 5 to 39) in KEYNOTE-028 trial, and only 5.8% in KEYNOTE-158 trial. As for nivolumab, its ORR reached 22% in BTC patients who had initially received 1–3 lines of treatment.^18^ There are also several ongoing trials regarding combination of immunotherapy and chemotherapy in BTC, such as pembrolizumab plus capecitabine/oxaliplatin (NCT03111732), pembrolizumab plus gemcitabine/cisplatin (NCT04003636), nivolumab plus gemcitabine plus cisplatin (NCT03101566) and so on, however, their results are not available yet. Although cross trial comparisons should be made cautiously, the ORR of camrelizumab plus GEMOX was observed to be much higher than immunotherapy alone, indicating that this is a promising strategy as first-line treatment of advanced BTC. Camrelizumab plus GEMOX was safe and tolerable. This toxicity profile was consistent with safety data reported for combination regimens in previous studies. Hypokalemia and fatigue were the most frequent grade 3–4 toxicities associated with camrelizumab combination therapy but were manageable with dose interruption/modification and appropriate supportive care. Of note, compared with the study by Fang *et al*,^19^ camrelizumab combination therapy in our study showed decreased incidence rates of grade 3–4 leukocytopenia (11% vs 48%), neutropenia (14% vs 57%) and thrombocytopenia (11% vs 31%). Although increase in the rate of certain toxicities with the use of triplet combination regimens might be inevitable like for instance vomiting (11% vs 0%), hypokalemia (3% vs 0%), increased GGT (14% vs 0%) and biliary tract infection (14% vs 0%). Potential immune-related adverse events such as increased aspartate aminotransferase, increased alanine aminotransferase and rash were generally manageable and reversible. Reactive cutaneous capillary endothelial proliferation was observed in almost two-thirds of patients in the trial and the incidence of RCCEP was similar to Fang’s study, but all were grade 1–2 and without significant effects on quality of life and medication. In addition, compared with GEMOX treatment alone,^20^ camrelizumab plus GEMOX showed decreased grade 3–4 adverse events (70% vs 84%). The current study showed that the safety profile of camrelizumab plus GEMOX was clinically acceptable in patients with advanced BTC.

PD-L1 expression on tumor cells, rather than on tumor infiltrating immune cells, was associated with response. A similar finding was obtained with anti PD-1 monotherapy whereby response was associated with expression of PD-L1 on tumor cells only (checkmate 017, 037; keynote 010), on the other hand, response with anti- PD-L1 monotherapy was associated with PD-L1 expression on both tumor cells and immune cells (POPLAR). However, the small number of events restricts the interpretation of these findings, and their clinical usefulness remains the subject of further study. Whether PD-L1 expression on tumor cells and tumor-infiltrating immune cells have a different predictive ability for camrelizumab plus GEMOX needs to be further studied.

In the present study, we did not observe any difference in the number or percentage of immune cells in center and in the invasive margin between responders and non-responders. Cytotoxic chemotherapy may enhance the antitumor response of PD-1/PD-L1 antibodies by increasing antigen cross-presentation by dendritic cells after the destruction of tumor cells, inhibiting myeloid-derived suppressor cells and increasing the ratio of cytotoxic lymphocytes to regulatory T cells. Diverse T cell infiltration increased in responders to nivolumab monotherapy in neoadjuvant setting (30297909). Thus, the dynamic change of tumor-infiltrating immune cells instead of baseline tumor-infiltrating immune cells is more likely to be associated with response to camrelizumab plus chemotherapy.

Tissue TMB or bTMB were not associated with response to camrelizumab plus GEMOX. These findings are consistent with those for NSCLC. Post-hoc analyzes and KEYNOTE 407 and 189 show that TMB, calculated by whole-exome sequencing, is not associated with any clinical benefit with pembrolizumab plus chemotherapy. Taken together, these results raise concerns whether TMB could serve as a predictive biomarker for PD-1/L1 antibodies plus chemotherapy.

Post-treatment ctDNA shows its potential to identify patients who respond to camrelizumab plus GEMOX. ORR was higher in patients with negative post-treatment ctDNA than in those with positive post-treatment ctDNA. These findings are consistent with previous retrospective studies of anti-PD-1/L1 monotherapy. In patients with melanoma treated with PD-1 inhibitors alone or in combination with ipilimumab, undetectable ctDNA within 12 weeks of therapy was associated with longer PFS and OS.^21^ In NSCLC treated with ICIs, a ctDNA response (defined as a>50% decrease in mutant allele fraction from baseline) was significantly earlier than radiographic response and was associated with improved patient survival.^22^ These findings support the potential utility of post-treatment ctDNA to monitor response of camrelizumab plus GEMOX in patients with BTC.

The interpretation of the findings may be limited by the relatively small sample size. However, the sample size was calculated under the assumption that a total of 35 patients would provide 80% power to detect a 6-month progression-free rate of 60% at a one-sided 2.5% alpha level under the null hypothesis of the 6-month progression-free rate equals to 40%. The efficacy and safety of camrelizumab plus GEMOX need to be further studied in a randomized controlled study with a larger sample size.

In summary, camrelizumab plus GEMOX as first-line treatment looks promising in patients with advanced BTC. The dynamic change of ctDNA might be an approach for the detection of response. Further randomized controlled studies will be needed in the future.

## Data Availability

All data relevant to the study are included in the article or uploaded as supplementary information. no.
