# Supplementary material for: Camrelizumab plus gemcitabine and oxaliplatin (GEMOX) in patients with advanced biliary tract cancer: a single-arm, open-label, phase II trial
Source: J Immunother Cancer. 2020 Nov 10;8(2):e001240. doi: 10.1136/jitc-2020-001240 (PMC7656907; doi:10.1136/jitc-2020-001240)
Supplement: Supplementary data [file jitc-2020-001240supp002.pdf]

Clinical Study Approval No.:

A Single-Arm, Open-Label, Exploratory Clinical Study of PD-1 Monoclonal Antibody SHR-1210 Combined with GEMOX (Gemcitabine Plus Oxaliplatin) in the Treatment of Advanced Biliary Tract Cancer

Study Protocol

Investigator-Initiated Trial (IIT)

**Protocol No.:**	**SHR1210-GEMOX-BTC-IIT03**
**Version No.:**	**V1.0**
**Version Date:**	**31 Aug., 2017**
**Principal Investigator:**	**Yongqian Shu**
**Sponsor:**	**Jiangsu Province Hospital**
**Funder:**	**Hengrui Medicine Co., Ltd.**<br>**Geneseeq Technology Inc.**

Confidentiality Statement

This document contains important confidential information. This information is proprietary to Shanghai Hengrui Pharmaceutical Co., Ltd./Jiangsu Hengrui Medicine Co., Ltd. and should not be disclosed unless required by current laws or regulations. In any case, all personnel receiving this document should be notified of this confidential requirement and should not further disclose this information.

Version History/Revision History

Document	Version Date	Amendment Rationale and Summary of Changes
V1.0	31 Aug., 2017	Not applicable

Principal Investigator's Signature Page

I will carefully execute the duties as an investigator in accordance with the Chinese GCP, and personally participate in or directly lead this clinical study. We have read and confirmed this protocol (protocol number: SHR-1210-GEMOX-BTC-IIT03, version number: V1.0, version date: 31 Aug., 2017). I agree to fulfill my duties in accordance with Chinese laws, the Declaration of Helsinki, the Chinese GCP, and this study protocol. Any modification to the protocol must be reviewed and approved by the sponsor, and can only be implemented upon approval by the Ethics Committee, unless measures must be taken to protect the safety, rights, and interests of the subjects.

STUDY SITE: JIANGSU PROVINCE HOSPITAL

Yongqian Shu		
Principal Investigator (print)	Principal Investigator (signature)	Signature Date (DD/MM/YYYY)

Chen X, *et al. J Immunother Cancer* 2022; 8:e001240. doi: 10.1136/jitc-2020-001240

Statistical Unit Signature Page

I will carefully execute the duties as a statistician in accordance with the provisions of the Chinese GCP and be responsible for the relevant statistics of this clinical study. We have read and confirmed this protocol (protocol number: SHR-1210-GEMOX-BTC-IIT03, version number: V1.0, version date: 31 Aug., 2017). I agree to fulfill my duties in accordance with Chinese laws, the Declaration of Helsinki, the Chinese GCP, and this study protocol. Any modification to the protocol must be reviewed and approved by the sponsor, and can only be implemented upon approval by the Ethics Committee, unless measures must be taken to protect the safety, rights, and interests of the subjects.

STATISTICAL UNIT:

Statistician (print)	Statistician (signature)	Signature Date (DD/MM/YYYY)

Chen X, *et al. J Immunother Cancer* 2022; 8:e001240. doi: 10.1136/jitc-2020-001240

TABLE OF CONTENTS

Version History/Revision History 2

PROTOCOL SYNOPSIS 10

SCHEDULE OF ACTIVITIES 15

ABBREVIATIONS AND DEFINITIONS 19

1. BACKGROUND 21

1.1 Epidemiology and Current Treatment of Biliary Tract Cancer 21

1.2 Developments in Targeted Therapy for BTC 22

1.3 Background of Immunotherapy 23

1.4 Development Background of PD-1 Antibody SHR-1210 24

1.5 Pre-Clinical Study Results of SHR-1210 25

1.5.1 Product name and physicochemical properties 25

1.5.2 Pharmacology and mechanism of action 25

1.5.3 Pharmacodynamic studies 25

1.5.4 Toxicology studies 27

1.5.5 Pharmacokinetic studies 27

1.5.6 Clinical study results 28

2. STUDY OBJECTIVES AND ENDPOINTS 28

2.1 Study Objectives 28

2.2 Primary Endpoints 28

2.3 Secondary Endpoints 28

2.4 Exploratory Endpoints 28

3. STUDY DESIGN 29

3.1 Overall Design 29

3.2 Dosing Regimen 30

3.2.1 Dose 30

Chen X, et al. J Immunother Cancer 2022; 8:e001240. doi: 10.1136/jitc-2020-001240

3.2.2 Method of administration and treatment cycles............30
3.3 Efficacy Evaluation and Analysis............30
3.4 Detection of Plasma Tumor Burden and Gene Mutation Changes............31
3.5 Follow-Up within 28 Days after Discontinuation or before the Start of New Anti-tumor Treatment............31
3.6 Follow-Up of AEs............31
3.7 Survival Follow-Up............31
4. RANDOMIZATION AND BLINDING............31
5. SELECTION AND WITHDRAWAL OF SUBJECTS............32
5.1 Inclusion Criteria............32
5.2 Exclusion Criteria............33
5.3 Study Withdrawal Criteria............34
5.4 Termination Criteria............34
6. STUDY DRUGS............35
6.1 Information............35
6.2 Labeling/Packaging............36
6.3 Drug Dispensation............36
6.4 Drug Storage and Management............36
6.5 Disposal of Remaining Drugs............36
6.6 Drug Preparation and Method of Administration............37
6.7 Concomitant Medication/Treatment............37
6.7.1 Drugs or treatment prohibited during the study............38
6.7.2 Medications and treatments that may be used concomitantly during the study............38
6.7.3 Recommended symptomatic treatment for common adverse reactions............39
7. DOSE MODIFICATION AND SAFETY MANAGEMENT............41
7.1 Dose Level and Use of Drugs............41

Chen X, *et al. J Immunother Cancer* 2022; 8:e001240. doi: 10.1136/jitc-2020-001240

7.2 Dose Modification 41

7.2.1 Criteria for dose modification...41

7.2.2 Criteria for dose delay 42

7.2.3 Criteria for resuming administration 43

7.2.4 Criteria for permanent discontinuation...43

7.2.5 Criteria for continuing treatment beyond progression..45

7.3 Safety Management for Immuno-Oncological Medications 46

7.3.1 Safety management procedures for immuno-oncological medications..........................46

7.3.2 Management procedures for hepatic AEs 47

8. STUDY PROCEDURES 47

8.1 Screening (from signing informed consent form to confirmed enrollment)......................47

8.2 Treatment Period...48

8.3 Follow-Ups 48

8.3.1 Follow-up of AEs..48

8.3.2 Survival follow-up 49

9. EFFICACY EVALUATION 49

9.1 Primary Endpoints and Observations 49

9.2 Secondary Endpoints and Observations 49

9.3 Exploratory Endpoints and Observations 50

10. SAFETY EVALUATION..50

10.1 Adverse Event (AE)...50

10.1.1 Definition of AE 50

10.1.2 AE severity grading criteria...50

10.1.3 Causality assessment 51

10.2 Serious Adverse Event (SAE)..52

10.2.1 Definition of SAE..52

10.2.2 Disease progression 52

10.2.3 Other anti-tumor treatments....................52

10.2.4 Hospitalization....................53

10.2.5 SAE reporting 54

10.2.6 Follow-up of SAEs 55

10.3 Pregnancy 55

10.4 AEs of Special Interest....................56

11. DATA MANAGEMENT....................56

11.1 Data Collection and Management....................56

11.1.1 Data collection 56

11.1.2 Data management and quality control 56

11.1.3 Data review and study site monitoring 57

12. DATA ANALYSIS AND STATISTICS 57

12.1 Determination of Sample Size....................57

12.2 Analysis Population 57

12.3 Statistical Analysis....................58

12.3.1 General analysis....................58

12.3.2 Efficacy analysis....................58

12.3.3 Safety analysis 59

12.3.4 Quality of life analysis....................59

12.3.5 Exploratory analysis 59

13. INFORMED CONSENT AND ETHICS....................59

13.1 Informed Consent 59

13.2 Ethics 59

13.3 Confidentiality of Subject Information....................60

14. DROP-OUTS....................60

Chen X, *et al. J Immunother Cancer* 2022; 8:e001240. doi: 10.1136/jitc-2020-001240

15. QUALITY CONTROL AND ASSURANCE 61
16. PUBLICATION OF STUDY RESULTS 61
17. TRIAL PROGRESS 61
18. TRIAL SITE AND TRIAL STAFF 61
Appendix 1. Response Evaluation Criteria in Solid Tumors 62
Appendix 2. Child-Pugh Score 77
Appendix 3. Performance Status (ECOG) 77
Appendix 4. New York Heart Association (NYHA) Functional Classification: Cardiac function is categorized into four classes, and heart failure is categorized into three degrees. 78

List of Tables

Table 1. Binding affinity of SHR-1210 to human, monkey, and rat PD-1 antigens. 25
Table 2. Binding affinity of SHR-1210, nivolumab, and MK3475 to PD-1 antigens. 26
Table 3. PK parameters after a single intravenous infusion at different doses of SHR-1210 in cynomolgus monkeys. 27
Table 4. Treatment recommendations for immune-related adverse reactions. 41
Table 5. Evaluation of causality between AEs and study drug. 51
Table 6. SAE report contacts. 54

List of Figures

Figure 1. Inhibition of PD-1/PD-L1 binding by SHR-1210 and pembrolizumab. 26
Figure 2. Inhibition of PD-1/PD-L1 binding by SHR-1210 and nivolumab. 26
Figure 3. Procedures of patient screening, treatment, and follow-up. 29

Chen X, *et al. J Immunother Cancer* 2022; 8:e001240. doi: 10.1136/jitc-2020-001240

PROTOCOL SYNOPSIS

Study Title	A Single-Arm, Open-Label, Exploratory Clinical Study of PD-1 Monoclonal Antibody SHR1210 Combined with GEMOX (Gemcitabine Plus Oxaliplatin) in the Treatment of Advanced Biliary Tract Cancer
Protocol No.	SHR1210-GEMOX-BTC-IIT03
Sponsor	Jiangsu Province Hospital
Study Population	Subjects with advanced biliary tract cancer (BTC)
Study Objectives	To observe and evaluate the efficacy and safety of PD-1 antibody SHR-1210 combined with GEMOX in patients with advanced BTC
Study Endpoints	Primary Endpoints:<br>1) 6-month progression-free survival (6-month PFS%)<br>2) Safety (no special safety events as assessed by the investigator)<br>Secondary Endpoints:<br>1) Objective response rate (ORR), as per ICR evaluation.<br>2) Duration of response (DoR), as per ICR evaluation.<br>3) Disease control rate (DCR), as per ICR evaluation.<br>4) 12-month overall survival rate (12-month OS)<br>5) Overall survival (OS)<br>Exploratory Endpoints:<br>1) PD-L1 expression<br>2) CD8+ TILs in tumor<br>3) Tumor mutation burden (TMB)<br>4) Plasma tumor mutation burden and changes of ctDNA amount
Study Design	This is a single-center, single-arm, open-label, prospective and exploratory clinical study.<br>**Subjects will receive treatments**:<br>In 28-day cycles.<br>D1 and D15: SHR-1210 3 mg/kg, up to 200 mg, I.V, q4w;<br>Gemcitabine 800 mg/m^2 IVD, q4w;<br>D2 and D16: Oxaliplatin 85 mg/m^2 IVD, q4w;<br>Subjects may receive combination chemotherapy for up to 6 cycles, and the cycles and doses of chemotherapy may be adjusted according to the patient's tolerability. Patients who are intolerant to chemotherapy or complete 6 cycles of chemotherapy but achieve stable disease or objective response will continue using PD-1 monoclonal antibody at 3 mg/kg for single-agent maintenance treatment with a total dose up to 200 mg/2w, until disease progression or unacceptable toxicity occurs or other situations judged by the investigator.

Chen X, *et al. J Immunother Cancer* 2022; 8:e001240. doi: 10.1136/jitc-2020-001240

Sample Size and Enrollment Plan	This study plans to enroll a total of 38 subjects.<br>Recruitment of new patients is expected to be completed within 12 months.
Screening Criteria	**Inclusion Criteria:**<br>1. Pathologically confirmed biliary tract cancer, including intrahepatic cholangiocarcinoma, extrahepatic cholangiocarcinoma, and gallbladder cancer.<br>2. Males or females aged 18-75 years old;<br>3. Expected survival ≥ 3 months;<br>4. ECOG PS score of 0-1;<br>5. Have at least 1 measurable lesion as per RECIST 1.1 criteria.<br>6. No previous treatment with oxaliplatin, gemcitabine, or PD-1/PD-L1 monoclonal antibodies.<br>7. Patients who have previously received tegafur or capecitabine as postoperative adjuvant chemotherapy or first-line treatment may be enrolled.<br>8. Able to provide enough tissue samples for PD-L1 immunohistochemical testing and second-generation sequencing;<br>9. Major organs function well, meeting the following criteria for the relevant examination indicators within 14 days before enrollment:<br>a) Hematology:<br>i. Hemoglobin ≥ 90 g/L (no blood transfusion within 14 days);<br>ii. Neutrophil count > 1.5×10^9/L;<br>iii. Platelet count ≥ 100×10^9/L;<br>b) Biochemistry:<br>i. Total bilirubin ≤ 1.5 × ULN (upper limit of normal);<br>ii. Blood alanine aminotransferase (ALT) or blood aspartate aminotransferase (AST) ≤ 2.5 × ULN; for those with liver metastases, ALT or AST ≤ 5 × ULN;<br>iii. Endogenous creatinine clearance ≥ 60 mL/min (Cockcroft-Gault formula);<br>c) Cardiac color Doppler: left ventricular ejection fraction (LVEF) ≥ 50%.<br>10. Signing informed consent form;<br>11. Subjects have good compliance and family members agree to corporate with the survival follow-up;<br>12. Female subjects: Have undergone surgical sterilization or be post-menopausal, or willing to take a medically approved contraceptive measure during and for 6 months after the study treatment; have a negative serum or urine pregnancy test within 7 days prior to enrollment, and not be breastfeeding. Male subjects: Have undergone surgical sterilization, or willing to take a medically approved contraceptive measure during and for 6 months after the study treatment.<br>**Exclusion Criteria:**

Chen X, et al. J Immunother Cancer 2022; 8:e001240. doi: 10.1136/jitc-2020-001240

	1. Have other previous or concomitant malignant tumors, excluding cured basal cell carcinoma and cervical carcinoma in situ; patients with other tumors such as concomitant small gastric stromal tumor that do not affect the patient's life in the short term may also be excluded.<br>2. Participation in other drug clinical trials within the last 4 weeks;<br>3. With dysphagia, chronic diarrhea, intestinal obstruction or other diseases affecting the nutritional status of patients;<br>4. Have a history of bleeding, and any Grade ≥ 3 bleeding as per CTCAE 4.0 within 4 weeks before screening;<br>5. Patients with known CNS metastasis or a history of CNS metastasis before screening. For patients with clinically suspected CNS metastasis, CT or MRI examinations must be performed within 28 days before enrollment to rule out CNS metastasis;<br>6. Patients with a history of unstable angina; newly diagnosed with angina within 3 months before screening or myocardial infarction within 6 months before screening; arrhythmia (including QTcF: ≥ 450 ms in males, ≥ 470 ms in females) that requires long-term use of antiarrhythmic drugs and New York Heart Association Class ≥ II cardiac insufficiency;<br>7. Urine protein ≥ ++ or 24-h urine protein > 1.0 g as indicated by urinalysis;<br>8. Have used immune-targeted drugs;<br>9. Have undergone liver transplant or other organ transplant;<br>10. Patients with infectious pneumonia, non-infectious pneumonia, interstitial pneumonia, or other diseases requiring corticosteroids;<br>11. Have a history of chronic autoimmune diseases, such as systemic lupus erythematosus;<br>12. Have a history of ulcerative colitis, Crohn's disease or other inflammatory bowel diseases, and a history of chronic diarrheal diseases such as irritable bowel syndrome;<br>13. Have a history of sarcoidosis or tuberculosis;<br>14. Patients with history of active hepatitis B and C, and HIV infection; patients with hepatitis B virus DNA controlled at < 2000 IU/mL may be enrolled.<br>15. Patients with high sensitivity to human or murine monoclonal antibodies;<br>16. Have a history of psychotropic substance abuse that unable to quit or have mental disorders;<br>17. Have clinical symptoms, pleural effusion or peritoneal effusion that require clinical intervention;<br>18. Have a history of immunodeficiency or other acquired or congenital immunodeficiencies;<br>19. Presence of accompanying diseases that may pose serious risks to the safety of the patient or may affect the patient's ability to complete the study as judged by the investigator;

	**Study Treatment Discontinuation Criteria:**<br>1. Subject withdraws informed consent and requests to withdraw from the study;<br>2. Imaging evaluations show disease progression, unless the subject meets the criteria for continuation of treatment beyond progression (see Section 7.2.5 for details);<br>3. Continuing participation in the study is not in the best interests of the subject due to clinical adverse reactions, laboratory abnormalities, or concurrent diseases, as assessed by the investigator;<br>4. Subject is required to withdraw for other investigator-assessed causes. For example, the subject can no longer express voluntary consent due to incarceration or quarantine;
Investigational Drug and Method of Administration	In 28-day cycles.<br>D1 and D15: SHR-1210 3 mg/kg, up to 200 mg, I.V, q4w;<br>Gemcitabine 800 mg/m^2 IVD, D1, D15, q4w;<br>D2 and D16: Oxaliplatin 85 mg/m^2 IVD, D2, D16, q4w;<br>Subjects may receive combination chemotherapy for up to 6 cycles, and the cycles and doses of chemotherapy may be adjusted according to the patient's tolerability. Patients who are intolerant to chemotherapy or complete 6 cycles of chemotherapy but achieve stable disease or objective response will continue using PD-1 monoclonal antibody at 3 mg/kg for single-agent maintenance treatment with a total dose up to 200 mg/2w, until disease progression or unacceptable toxicity occurs or other situations judged by the investigator.
Statistical Methods	**General Analysis**<br>Unless otherwise stated, all experimental data in this study will be summarized with descriptive statistics according to the following general principles.<br>Continuous variables will be summarized by mean, standard deviation, median, maximum, and minimum; categorical variables will be summarized by frequencies and percentages; for time-to-event data, the survival rate and median survival will be estimated using the Kaplan-Meier method.<br>**Efficacy Analysis**<br>The survival distribution of time-event variables (including OS, TTP, and PFS) will be estimated using the Kaplan-Meier method and the median and two-sided 95% confidence interval (CI) will be calculated. The objective response rate (ORR) and disease control rate (DCR) and their 95% CIs will be calculated using the Clopper-Pearson method.<br>**Safety Analysis**<br>AEs, SAEs, treatment-related AEs, AEs resulting in dose modification, AEs resulting in withdrawal, and laboratory data will be summarized statistically according to CTCAE 4.0. The safety analysis is mainly based on descriptive statistics.<br>**Exploratory Analysis**<br>PD-L1 expression and CD8+ TILs will be analyzed based on descriptive statistics.<br>Tissue TMB analysis, blood TMB and changes in ctDNA amount before and 2 months after treatment

	**Sample Size Determination**<br>In previous clinical studies of gemcitabine combined with oxaliplatin as first-line treatment of advanced BTC, the median survival of patients was 3-5.5 months, and the 6-month PFS was 30-40%. Of these reports, two were from East Asian populations (Korea) with a 6-month PFS of approximately 30%. In the BINGO study, the 6-month PFS of the GEMOX group was approximately 40%. With reference to these studies, assuming a 6-month PFS of 40% in the first-line treatment of gemcitabine combined with oxaliplatin in patients with advanced BTC in China, the 6-month PFS in this study is expected to be increased to 60%; with one-sided test, alpha of 0.05, and power of 80%, a sample size of 35 subjects is required. Considering a drop-out rate (within 10%) in study visits, 38 subjects need to be enrolled.
Version No.	V1.0
Study Period	Oct. 2017 to Apr. 2020

SCHEDULE OF ACTIVITIES

Items	Screening Period [1] (-28d)	Cycle 1[2]		Cycle 2		Cycle 3		Cycles 4-6		End of Treatment[3]	Follow-Up Period	
		D1 (± 3d)	D15 (± 3d)	D1 (± 3d)	D15 (± 3d)	D1 (± 3d)	D15 (± 3d)	D1 (± 3d)	D15 (±3 d)		Safety follow-up[4]	Survival follow-up[5]
Signing Informed Consent	√											
Demographics	√											
Medical History	√											
**ECOG** PS Score	√	√	√	√	√	√	√	√	√	√	√	
Physical Examination[6]	√	√	√	√	√	√	√	√	√	√	√	
Vital Signs[7]	√	√	√	√	√	√	√	√	√	√	√	
Hematology[8]	√	√	√	√	√	√	√	√	√	√	√	
Urinalysis[9]	√	√	√	√	√	√	√	√	√	√	√	
Fecal Occult Blood[10]	√											
Blood Biochemistry[11]	√	√	√	√	√	√	√	√	√	√	√	
Blood Electrolytes[12]	√	√	√	√	√	√	√	√	√	√	√	
Coagulation Function[13]	√			√		√		√		√	√	
Child-Pugh Score	√					√				√	√	
Carbohydrate Antigen 199[14]	√			√		√		√		√	√	

Items	Screening Period [1] (-28d)	Cycle 1[2]		Cycle 2		Cycle 3		Cycles 4-6		End of Treatment[3]	Follow-Up Period	
		D1 (± 3d)	D15 (± 3d)	D1 (± 3d)	D15 (± 3d)	D1 (± 3d)	D15 (± 3d)	D1 (± 3d)	D15 (±3 d)		Safety follow-up[4]	Survival follow-up[5]
Carcinoembryonic Antigen[15]	√			√		√		√		√	√	
Thyroid Function[16]	√					√				√	√	
ECG[17]	√									√		
Echocardiography[18]	√									√		
Tumor Imaging Evaluation[19]	√					√				√		
Detection of Plasma Tumor Molecular Burden and Gene Mutation[20]	√					√						
Hepatitis B, Hepatitis C and HIV Tests[21]	√											
Tumor Tissue Sample[22]	√											
Blood HCG Test[23]	√									√		
Concomitant Medication/ Treatment[24]	√											
Adverse Events[25]	√	√	√	√	√	√	√	√	√	√	√	

Chen X, *et al. J Immunother Cancer* 2022; 8:e001240. doi: 10.1136/jitc-2020-001240

Items	Screening Period [1] (-28d)	Cycle 1[2]		Cycle 2		Cycle 3		Cycles 4-6		End of Treatment[3]	Follow-Up Period	
		D1 (± 3d)	D15 (± 3d)	D1 (± 3d)	D15 (± 3d)	D1 (± 3d)	D15 (± 3d)	D1 (± 3d)	D15 (±3 d)		Safety follow-up[4]	Survival follow-up[5]
Administration of SHR-1210 Combined with Chemotherapy		**D1 and D15**: SHR-1210 3 mg/kg, up to 200 mg, I.V, q4w;<br>Gemcitabine 800 mg/m^2 IVD, D1, D15, q4w;<br>**D2 and D16**: Oxaliplatin 85 mg/m^2 IVD, D2, D16, q4w;<br>**After Cycle 6**: SHR-1210 3 mg/kg, up to 200 mg, I.V, q2w										

Note: In addition to the examinations and time points shown in this table, the investigator may order additional tests at any time. Test results should be entered into the "Unscheduled Tests" sheet of the CRF. (Deleted or added as needed)

[1] Screening period: Physical examination, vital signs, and laboratory tests (including hematology, urinalysis, fecal occult blood, hepatic and renal function, blood electrolytes, blood glucose, LDH, amylase/lipase <repeat lipase test when amylase is abnormal>, coagulation function, thyroid function, and virological examination) should be performed within 14 days before the start of study treatment; tumor imaging evaluations (thoracic, abdominal, pelvic, and brain CT/MRI scans) and echocardiography of a subject that meet the requirements within 28 days before the start of study treatment do not need to be repeated.

[2] Study treatments are in cycles of 28 days.

[3] End of study treatment/withdrawal: These examinations do not need to be repeated in this period if they have been completed within 7 days from withdrawal.

[4] Safety follow-up: 28 days after the last dose.

[5] Survival follow-up: Follow-up via telephone once per month to collect information of survival and subsequent treatments.

[6] Physical examination: A complete physical examination should be performed at screening or before the Cycle 1 administration, and then a targeted examination should be performed before each administration, including at least the cardiovascular, digestive, and respiratory systems, and skin.

[7] Vital signs: Including body temperature, blood pressure, heart rate, and respiratory rate, examined at screening, within 7 days before the first study treatment, and before each subsequent administration.

[8] Hematology: Tested once within 72 h before each administration, including complete blood count with differential (white blood cells, red blood cells, lymphocytes, monocytes, neutrophils, basophils, eosinophils, and hemoglobin), and platelet count. The study medication can only be given after reviewing the results.

[9] Urinalysis: In case of a urine protein ≥ 2+ at screening, a 24-h urine protein quantitation should be added; during the study period, the urinalysis should be performed within 72 h before each administration, and in case of a urine protein ≥ 2+ at screening, a 24-h urine protein quantitation should be added.

[10] Fecal occult blood: Tested only when clinically indicated at screening and during the study period.

[11] Blood biochemistry: Examined once within 72 h before each administration, including hepatic function (ALT, AST, total bilirubin, and ALP), renal function (urea nitrogen or urea, creatinine), albumin, blood glucose, LDH, amylase (add lipase test if amylase is abnormal). The study medication can only be given after reviewing the results.

[12] Blood electrolytes: Tested once within 72 h before each administration, including Ca^{2+}, Mg^{2+}, Na^{+}, K^{+}, and Cl^{-}.

[13] Coagulation function: Tested once at screening and on D1 ± 1 of each cycle after Cycle 2, including the international normalized ratio (INR); if INR is not available, prothrombin time (PT) can be used instead.

[14] Carbohydrate antigen 199: Tested once at screening and on D1 ± 1 of each cycle after Cycle 2.

[15] Carcinoembryonic antigen: Tested once at screening and on D1 ± 1 of each cycle after Cycle 2.

[16] Thyroid function: TSH, fT3, fT4 (or T3, T4) are tested at screening, and TSH is tested once on D1 ± 1 of each cycle after the start of Cycle 2 (fT3/fT4 or T3/T4 should be retested if TSH is abnormal).

[17] ECG: 12-lead ECG. At screening and at the end of treatment. Performed according to local standard practice when clinically indicated.

[18] Echocardiography: At screening and at the end of treatment. Performed according to local standard practice when clinically indicated.

[19] Tumor imaging evaluation: Performed as per RECIST 1.1 criteria. The baseline (screening period) imaging evaluation must be performed within 28 days before the start of study treatment (enhanced CT of the chest, abdomen, and pelvis, and CT/MRI scans of the brain). A bone scan should be performed when clinically indicated, and must be within 42 days. The first tumor evaluation is performed at Week 8 (D56 ± 7) after the start of study treatment, and repeated every 8 weeks thereafter, until disease progression or the end of treatment (whichever occurs later), regardless of any treatment delay. Subjects with brain metastases are recommended to monitor brain metastases with MRI scans. MRI is repeated approximately every 12 weeks from the start of study treatment. The frequency of monitoring may be increased when clinically indicated.

[20] Detection of plasma tumor molecular burden and gene mutation: Corresponding to imaging examinations. To explore the changes of plasma tumor molecular burden with treatment, the corresponding relationship with imaging changes, and the changes in mutation spectrum.

[21] Hepatitis B, hepatitis C and HIV tests: If hepatitis B surface antigen is tested positive, virus DNA needs to be tested.

[22] Tumor tissue sample: Used for exploratory detection of PD-L1 expression, CD8+ TILs, and TMB. See the laboratory manual.

[23] Blood HCG test: Only for women of childbearing potential, tested within 72 h before the first dose and at the end-of-study visit.

[24] Concomitant medication/treatment: Documentation is required for each visit; subsequent anti-tumor treatments should be recorded during the follow-up period.

[25] Adverse events: Adverse events are recorded until 90 days after the last dose.

ABBREVIATIONS AND DEFINITIONS

The following abbreviations and special terms are used in this study protocol

Abbreviations and Special Terms	Definitions
AE	Adverse event
AFP	Alpha-fetoprotein
AKP	Alkaline phosphatase
ALT	Alanine aminotransferase
ANC	Absolute neutrophil count
APTT	Activated partial thromboplastin time
AST	Aspartate aminotransferase
BUN	Blood urea nitrogen
B-Scan	B mode ultrasound
Ca	Calcium
CA19-9	Carbohydrate antigen 199
Cl	Chlorine
CEA	Carcinoembryonic antigen
Cr	Creatinine
CR	Complete response
CT	Computed tomography
DCR	Disease control rate
DLT	Dose-limiting toxicity
ECOG PS	Eastern Cooperative Oncology Group Performance Status
eCRF	Electronic case report form
GCP	Good Clinical Practice
FAS	Full analysis set
Glu	Glucose
Hb	Hemoglobin
HCC	Hepatocellular carcinoma
ITT	Intent-to-treat
K	Potassium
LD50	Median lethal dose
MRI	Magnetic resonance imaging
MTD	Maximum tolerated dose
Na	Sodium
NCI-CTC	National Cancer Institute Common Toxicity Criteria
OB	Occult blood

Chen X, *et al. J Immunother Cancer* 2022; 8:e001240. doi: 10.1136/jitc-2020-001240

Abbreviations and Special Terms	Definitions
ORR	Objective response rate
OS	Overall survival
PD	Progressive disease
PFS	Progression-free survival
PI	Principal Investigator
PLT	Platelet
PR	Partial response
PT	Prothrombin time
q.d.	Quaque die
QoL	Quality of life
RBC	Red blood cell
RC	Red color sign
RECIST	Response evaluation criteria in solid tumors
RR	Response rate
RTKs	Receptor tyrosine kinases
γ-GT	Gamma-glutamyltransferase
SAE	Serious adverse event
SAS	Safety analysis set
SD	Stable disease
TMB	Tumor mutation burden
TT	Thrombin time
TTP	Time to progression
ULN	Upper limit of normal
UPC	Urine protein concentration
URIC	Uric acid
WBC	White blood cell

1. BACKGROUND

1.1 Epidemiology and Current Treatment of Biliary Tract Cancer

Biliary tract cancer (BTC) accounts for 4% of digestive system malignancies. Its incidence varies widely by gender, race, and region. BTC is one of the most common causes of cancer mortality in Chile or northern India, with a ratio of 1.5 in male to female and a median age of onset of 70 years old. Data on BTC are lacking in China. Results from some registered regions show that the incidence of gallbladder cancer accounts for 1.7% of all tumors and the mortality is 1.9% (Globocan 2012). Surgical resection is the only possible way to cure BTC. However, the reported resection rates vary widely depending on the primary site. Most BTC patients cannot obtain a radical resection at the time of diagnosis, and up to 70% of the patients have positive margin (R1) under microscope. The positive rate is 70% in ampullary tumor, 40-50% in gallbladder cancer, and 30% in intrahepatic cholangiocarcinoma and distal extrahepatic cholangiocarcinoma. The prognosis after resection varies greatly depending on the anatomical site of the tumor. The 5-year survival rate is 20-40% for intrahepatic cholangiocarcinoma, 50-70% for ampullary tumor, 25-50% for distal cholangiocarcinoma and gallbladder cancer, and 15-35% for hilar cholangiocarcinoma. Prognostic factors that have been identified include tumor staging, lymph node status, histological grade, and marginal status. These factors are widely used in prospective studies to stratify patients. Other prognostic factors are still controversial, such as CA199, vascular and perineural invasion.

Abnormalities in multiple genes are common in BTC, especially genetic abnormalities in the EGFR signaling pathway, including EGFR, KRAS, BRAF, PI3KCA, etc. Mutation rates vary widely across studies and in different sites of the biliary tract. It is worth noting that in intrahepatic bile duct cancer, the KRAS mutation rate may be as high as 54% (Table 1). A study (2011) in China showed that 38.2% and 32.4% of KRAS mutations and PI3KCA mutations, respectively, were found in bile duct cancer by sequencing, but no BRAF mutations were detected. A Chinese-based study recently published in Nature Gentics in 2014 showed that the mutation rates of RAS (including KRAS, NRAS, and HRAS), BRAF, and PI3KCA detected in patients with gallbladder cancer using whole-exome sequencing and target gene sequencing were 11.8%, 5.9%, and 5.9%, respectively. In addition, there are still many other genetic abnormalities in the EGFR signaling pathway. However, sensitive mutations in EGFR tyrosine kinase inhibitors (TKIs), such as L858R point mutations of exons Del 19 and 21, are almost absent in BTC. For unresectable and metastatic BTC, palliative chemotherapy is an option that may provide survival benefits. A randomized controlled phase III study (ABC-02 trial) published in NEJM in 2010 showed that, compared with gemcitabine monotherapy, gemcitabine combined with cisplatin extended the median survival of patients from 8.1 months to 11.7 months (HR: 0.64, 95% CI: 0.52-0.80, $P < 0.001$). In addition to more neutropenia in the combination treatment group, adverse events were similar in the two

Supplemental material BMJ Publishing Group Limited (BMJ) disclaims all liability and responsibility arising from any reliance placed on this supplemental material which has been supplied by the author(s) J Immunother Cancer

groups, and the proportion of infections due to neutropenia was similar in the two groups. Another phase III clinical study published in JCO in 2010 showed that gemcitabine combined with oxaliplatin (GemOX) significantly prolonged survival compared with the best supportive care group (HR: 0.44; 95% CI: 0.22 to 0.86, p = 0.01). Oxaliplatin is more commonly used in the treatment of BTC than cisplatin in the clinical practice and has better safety. Also, more clinical studies have confirmed that gemcitabine combined with oxaliplatin can provide patients with higher response rate and survival. These studies established the standard first-line chemotherapy regimen for advanced BTC with 6-8 cycles of gemcitabine combined with platinum-based treatment.

1.2 Developments in Targeted Therapy for BTC

In view of the success of anti-EGFR monoclonal antibodies and anti-VEGF monoclonal antibodies in the treatment of colorectal cancer, investigators have also tried to use chemotherapy combined with anti-EGFR monoclonal antibodies (cetuximab and panitumumab) or bevacizumab for the treatment of BTC.

The BINGO study, published in Lancet Oncol in 2014, is a randomized controlled, open-label, non-comparative phase II clinical study [19]. Seventy-four (74) patients received GEMOX chemotherapy alone, and 76 patients received GEMOX combined with cetuximab. KRAS status was not an inclusion criterion for patients, and only exploratory analysis was performed at a later stage. The median values of PFS in the two groups were 5.5 months and 6.1 months, respectively. The median values of OS were 11.0 and 12.4 months, respectively. Toxicity was well tolerated in both groups. Cetuximab showed some advantages in survival. The main limitation of this study, as the authors wrote, is that the 150 patients designed to be enrolled are insufficient to support a formal comparison, thus a non-comparative study design was used. The number of subjects in other studies of anti-EGFR monoclonal antibodies combined with chemotherapy is mostly under 50, and it is difficult to obtain objective and reliable poor-phase data.

Chemotherapy combined with bevacizumab has also been attempted in the treatment of BTC. However, all existing studies had negative results. Therefore, in the palliative treatment of BTC, the combination of chemotherapy and targeted therapy is not currently recommended.

Chen X, *et al. J Immunother Cancer* 2022; 8:e001240. doi: 10.1136/jitc-2020-001240

1.3 Background of Immunotherapy

Cancer immunotherapy is a long-time hot spot in the field of cancer treatment, in which the cancer immunotherapy using T cells is at the core position. Cancer immunotherapy fully utilizes and mobilizes killer T cells in cancer patients to kill tumors, and may be the most effective and safest way to treat cancer. Also, tumor immune escape is a great challenge in cancer immunotherapy. Cancer cells' suppressive effect on the immune system promotes uncontrolled tumor growth. There is an extremely complex relationship between the immune escape mechanism of tumors and the body's anti-tumor immune response. Tumor-specific killer T cells have certain biological activities in the early stage of the cancer immunotherapy, but they lose their cytotoxicity in the late stage of tumor growth. So the cancer immunotherapy maximizes a patient's own immune response to the tumor, by not only activating the body's original immune response, but also maintaining the duration and intensity of the immune response which is the key to immunotherapy.

Recently, with better understanding of the mechanism of controlling host response to tumor at the molecular level, the identification of signaling pathways that restricts anti-cancer immune response has been further promoted. The programmed cell death protein-1 (PD-1) pathway, one of the most critical checkpoint pathways responsible for regulating tumor-induced immunosuppression, has been substantiated. Programmed death-1 (PD-1) is a protein receptor expressed on the surface of T cells discovered in 1992 and is involved in the process of cell apoptosis. PD-1 is a member of the CD28 family and has a 23% consistency in amino acid sequence with cytotoxic T lymphocyte antigen 4 (CTLA-4). However, its expression is different from CTLA. It is primarily expressed by activated T cells, B cells, and myeloid cells. PD-1 has two ligands, PD-L1 and PD-L2. PD-L1 is primarily expressed on T cells, B cells, macrophages, and dendritic cells (DCs), and is up-regulated on activated cells. Many human solid tumors have expression of the PD-1 ligand (PD-L1), which is often associated with a poor prognosis. Tumor-infiltrating lymphocytes in patients with cancers usually express PD-1 and their anti-tumor function is impaired. Pre-clinical trials of several antibodies that block PD-1 or PD-L1 have proven that they can enhance T-cell function and promote tumor cell lysis.

A number of multinational pharmaceutical companies are currently developing monoclonal antibodies targeting PD-1. By blocking the binding of PD-L1/PD-1, the patient's own immune response to the tumor is maximized to kill the cancer cells and cause apoptosis. BMS and Merck's PD-1 monoclonal antibodies are currently the most advanced PD-1 antibodies. Based on the results of key studies that have been completed, the FDA has approved nivolumab from BMS and pembrolizumab from Merck for multiple indications.

In July 2014, nivoluamb was approved by the Japanese Ministry of Health and Welfare for the treatment of advanced melanoma. It was approved by the FDA for the treatment of melanoma in December 2014, approved by the FDA for the treatment of non-small cell lung cancer in March 2015, and approved by the FDA for the treatment of renal cell carcinoma in November 2015. In 2016, indications of nivoluamb were added by its approval for use in the treatment of classical Hodgkin's lymphoma.

In September 2014, Pembrolizumab was approved by the FDA for the treatment of advanced melanoma. In October 2015, Pembrolizumab was approved by the FDA for the treatment of non-small cell lung cancer.

In addition to the above indications, the two companies are conducting large-scale phase III studies in the fields of gastric cancer, bladder cancer, breast cancer, head and neck tumors, and lymphoma. It is expected that the treatment of multiple tumors will bring new options and impacts.

In 2015 ESMO, mid-term data from the keynote 028 series of studies of biliary tract cancer were published (abstract no.: 525). The study enrolled 24 patients with PD-L1-positive biliary tract cancer, used second-line and above treatment with pembrolizumab, and found that 4 subjects achieved objective response, 4 subjects had stable disease, and 12 subjects experienced disease progression, with no survival data reported. These data suggested that immunotherapy had certain efficacy in patients with biliary tract cancer, but the efficacy was low. In this study, enrollments were PD-L1 positive patients. It is unknown whether PD-L1 negative patients will benefit. For lung cancer, chemotherapy combined with immunotherapy shows higher activity and may also be effective in PD-L1 negative patients. Then, for biliary tract cancer, can chemotherapy combined with immunotherapy further improve the efficacy and PFS? Can PD-L1 be a predictive marker of efficacy for immunotherapy of biliary tract cancer? Are there any other biomarkers, including gene mutation, tumor mutation burden, and immune microenvironment changes, that can be used as predictive markers of efficacy for immunotherapy of biliary tract cancer?

1.4 Development Background of PD-1 Antibody SHR-1210

Using PD-1 as the target, Hengrui obtained a series of murine anti-PD-1 antibodies by using recombinant PD-1 proteins as the immunogen. Through a large number of in vitro binding assays, in vitro ligand blocking assays, T cell proliferation assays, animal experiments and druggability assessments, an antibody prototype was selected. Then, a humanized design of the murine antibody prototype was carried out through computer simulations, resulting in several humanized anti-PD-1 monoclonal antibodies. Finally, SHR-1210, the antibody with the highest activity, was selected for further development. Phase I clinical studies have been conducted by Hengrui in Australia and China since 2015. Several phase II-III clinical studies are currently underway.

1.5 Pre-Clinical Study Results of SHR-1210

1.5.1 Product name and physicochemical properties

[Generic Name]: SHR-1210 Injection

[English Name]: SHR-1210 Injection

[Molecular Weight]: approx. 146.3 kDa

1.5.2 Pharmacology and mechanism of action

Programmed death-1 (PD-1) is a protein receptor expressed on the surface of T cells discovered in 1992 and is involved in the process of cell apoptosis. PD-1 is a member of the CD28 family and has a 23% consistency in amino acid sequence with cytotoxic T lymphocyte antigen 4 (CTLA-4). However, its expression is different from that of CTLA. It is primarily expressed by activated T cells, B cells, and myeloid cells. PD-1 has two ligands, PD-L1 and PD-L2. PD-L1 is primarily expressed on T cells, B cells, macrophages, and dendritic cells (DCs), and is up-regulated on activated cells. In contrast, the expression of PD-L2 is mainly restricted to antigen presenting cells, such as activated macrophages and DCs.

Humanized anti-PD-1 monoclonal antibody can specifically bind to PD-1, blocking the interaction between PD-1 and its ligands, and restore T cell immune response to tumor cells.

1.5.3 Pharmacodynamic studies

1.5.3.1 Antibody affinity

The binding affinity of antibody SHR-1210 to human, monkey and rat antigens was assayed. The results are shown in Table 1.

Table 1. Binding affinity of SHR-1210 to human, monkey, and rat PD-1 antigens.

Stationary Phase	Mobile Phase	Affinity (nM)
SHR-1210	Human PD-1 antigen	6.9
SHR-1210	Rat PD-1 antigen	Extremely weak signals, no binding detected
Monkey PD-1 antigen (-hFc)	SHR-1210	4.1

Results from affinity assays showed that the affinities of SHR-1210 to human and monkey PD-1 antigens were 6.9 nM and 4.1 nM, respectively, but no binding was detected with rat PD-1 antigens.

Chen X, *et al. J Immunother Cancer* 2022; 8:e001240. doi: 10.1136/jitc-2020-001240

Results from the binding affinity assay involving SHR-1210 and human PD-1 antigen showed that the binding affinity of SHR-1210 to human PD-1 antigen was 3.0 nM, which was similar to those of the control antibodies nivolumab and MK3475. The results are shown in Table 2.

Table 2. Binding affinity of SHR-1210, nivolumab, and MK3475 to PD-1 antigens.

Antibody	Antigen	Affinity (nM)
SHR-1210	Human PD-1 antigen	3.0
Nivolumab	Human PD-1 antigen	4.0
MK3475	Human PD-1 antigen	3.2

1.5.3.2 Inhibition of PD-1/PD-L1 binding by SHR-1210

Experiment results (see Figure 1, Figure 2) showed that in vitro inhibition activity of SHR-1210 on PD-1/PD-L1 binding was similar to nivolumab and pembrolizumab.The IC_{50} of inhibition activities of SHR-1210, nivolumab and pembrolizumab were 0.70 nM/0.79 nM and 0.79 nM/0.77 nM, respectively.

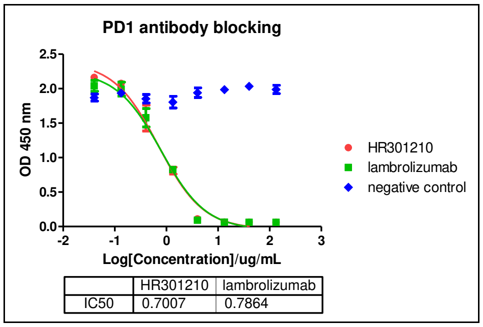

Figure 1. Inhibition of PD-1/PD-L1 binding by SHR-1210 and pembrolizumab.

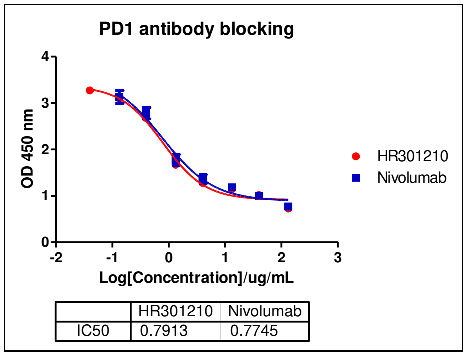

Figure 2. Inhibition of PD-1/PD-L1 binding by SHR-1210 and nivolumab.

1.5.4 Toxicology studies

In an acute toxicity study in cynomolgus monkeys, 8 monkeys (half male and half female) were randomized to 2 groups. The animals in Group 2 were given an intravenous injection of SHR-1210 once every other day at doses of 200, 400 and 800 mg/kg, respectively, in a dose escalation manner. No changes in clinical symptoms, weight, food intake, and coagulation related to SHR-1210 were observed. Lymphocytes decreased for both sexes at doses ≥ 200 mg/kg. Serum globulin increased and albumin decreased at doses ≥ 400 mg/kg. Since the magnitude of these changes were small, they were not considered harmful effects. The maximum tolerated dose (MTD) of SHR-1210 was ≥ 800 mg/kg.

In a completed preclinical long term toxicity study in cynomolgus monkeys, continuous intravenous administration of SHR-1210 at 20, 50, and 100 mg/kg/dose for 4 weeks (5 total doses) were well-tolerated in both sexes. Clinical symptoms, including injection site irritation, or changes in body weight, food intake, body temperature, ECG, blood pressure, heart rate and respiratory measurements related to SHR-1210 were not observed. No changes in B and T cell differentiation, cytokines, immunoglobulins, and complements were observed. No changes in organ weight, gross lesions, or histopathological changes associated with SHR-1210 were observed.

1.5.5 Pharmacokinetic studies

For SHR-1210 PK parameters after a single intravenous infusion in cynomolgus monkeys, see Table 3.

Table 3. PK parameters after a single intravenous infusion at different doses of SHR-1210 in cynomolgus monkeys.

Dose (mg/kg)	Gender	$T_{1/2}$ (hr)	T_{max} (hr)	C_{max} (ug/mL)	AUC_{last} (hr*ug/mL)	Vz (mL/kg)	Cl (mL/hr/kg)	MRT_{last} (hr)
1	Female	76.06±32.93	0.83±0.29	31.16±11.25	1716.12±453	54.09±14.85	0.57±0.17	80.95±18.58
	Male	91.72±25.26	0.83±0.29	35.96±13.09	2359.7±684.07	55.15±20.51	0.37±0.06	102.23±38.56
	Overall	83.89 ±27.62	0.83 ±0.26	33.56± 11.23	2037.91± 627.32	54.62± 16.02	0.47± 0.15	91.59 ±29.47
3	Female	92.95±22.60	0.83±0.29	81.09±12.66	6896.79±1673.36	40.75±12.66	0.44±0.11	120.92±49.96
	Male	113.54±8.26	1.67±0.58	71.65±10.85	6380.25±2062.85	47.05±27.05	0.47±0.12	127.10±59.25
	Overall	103.25±18.94	1.25 ±0.61	76.37 ±11.74	6638.51 ±1703.60	43.91 ±19.21	0.46 ±0.11	125.01±49.13
10	Female	169.70±38.96	2.17±1.76	217.46±20.22	31357.28±9338.28	41.25±25.76	0.33±0.1	179.68±73.6
	Male	128.94±35.93	0.67±0.29	251.88±6.49	26779.98±7205.43	30.9±30.2	0.31±0.05	113.25±44.39
	Overall	149.32±40.28	1.42±1.39	234.67±23.15	29068.63±7869.83	36.07±25.34	0.32±0.07	146.46±65.42

Chen X, *et al. J Immunother Cancer* 2022; 8:e001240. doi: 10.1136/jitc-2020-001240

1.5.6 Clinical study results

In 2015, Hengrui successfully conducted a phase I clinical trial in Australia and obtained the expected clinical results (see IB). Phase I clinical trials (SHR-1210-101, SHR-1210-102, and SHR-1210-103) and phase II-III trials of SHR-1210 have also been initiated in multiple clinical sites in China. More clinical data will be obtained.

2. STUDY OBJECTIVES AND ENDPOINTS

2.1 Study Objectives

To observe and evaluate the efficacy and safety of PD-1 antibody SHR-1210 combined with GEMOX in patients with advanced BTC

2.2 Primary Endpoints

6-month progression-free survival (6-month PFS%)

Safety

2.3 Secondary Endpoints

Objective response rate (ORR), as per ICR evaluation.

Duration of response (DoR), as per ICR evaluation.

Disease control rate (DCR), as per ICR evaluation.

12-month survival

Overall survival (OS)

2.4 Exploratory Endpoints

PD-L1 expression

CD8+ TILs in tumor

Tumor mutation burden (TMB)

Blood tumor mutation burden and changes of ctDNA amout before and two months after treatment

3. STUDY DESIGN

3.1 Overall Design

This is a single-center, single-arm, open-label, prospective and exploratory clinical study.

Study procedures are shown below:

Figure 3. Procedures of patient screening, treatment, and follow-up.

Screening: Carried out within 4 weeks prior to the first dose

↓

- The investigator should confirm the medical history data of subjects
- Obtain histopathological specimens and baseline plasma specimens of the subject
- Log in to the system and enter the information
- Obtain study number and drug number

↓

Treatment and efficacy evaluation:

- Until imaging confirmed PD or other situations resulting in discontinuation
- Efficacy evaluation: The first efficacy evaluation is performed at Week 8 (± 7 days) after administration, and once every 8 weeks (± 7 days) thereafter
- At each imaging evaluation (± 2 days), blood samples are collected for detection of plasma tumor DNA and mutation
- Safety evaluation: Conducted every cycle; objective endpoints are obtained through inspection, and subjective endpoints are obtained through patient-reported adverse events.

↓

Follow-up: Starts immediately after withdrawing from study treatment

- Mandatory safety follow-up is conducted approximately 28 days after the last dose of study treatment or before the start of new anti-tumor treatment (whichever occurs first). Document all adverse events that occur before the safety follow-up visit.
- Thereafter, survival follow-up is conducted once per month by telephone

Supplemental material BMJ Publishing Group Limited (BMJ) disclaims all liability and responsibility arising from any reliance placed on this supplemental material which has been supplied by the author(s) J Immunother Cancer

If there are no such conditions as the subject's withdrawal of informed consent form, unacceptable toxicity, or other situations that the investigator considers not suitable for further trials, each subject is expected to receive up to 6 cycles of combination chemotherapy, with the treatment with SHR-1210 continued until imaging confirmed tumor progression or for up to 2 years. The efficacy and safety endpoints should be monitored during the trial.

After the 28-day follow-up necessary for treatment discontinuation, the subjects will continue to be followed to gather information on their survival.

3.2 Dosing Regimen

3.2.1 Dose

Gemcitabine 800 mg/m^2 IVD, D1, D15, q2w

Oxaliplatin 85 mg/m^2 IVD, D2, D16, q2w

SHR-1210 3 mg/kg, with the total dose up to 200 mg, I.V, D1, D15, q2w, in 28-day cycles.

3.2.2 Method of administration and treatment cycles

Chemotherapy combined with SHR-1210 is given via intravenous injection at corresponding doses in 28-day cycles. Subjects may receive combination chemotherapy for up to 6 cycles, and the cycles and doses of chemotherapy may be adjusted according to the patient's tolerability. Patients who are intolerant to chemotherapy or complete 6 cycles of chemotherapy but achieve stable disease or objective response will continue using PD-1 monoclonal antibody at 3 mg/kg for single-agent maintenance treatment with a total dose up to 200 mg/2w, until disease progression or unacceptable toxicity occurs or other situations judged by the investigator.

Dose modifications for chemotherapy and SHR-1210 are described later.

3.3 Efficacy Evaluation and Analysis

The first tumor imaging evaluation after the start of study treatment is performed in Week 8 (D56 ± 7 days); efficacy evaluations are performed once every 8 weeks (based on calendar days, not affected by drug discontinuation) thereafter, and within ± 7 days (except for statutory holidays) at the end of every 8 weeks. CT or MRI scans are needed for evaluations. The radiographic technique should be consistent for all the tests of a subject, and all the images should be retained.

Any doubt about radiographic disease progression may be confirmed by the investigator during subsequent tumor evaluations.

Chen X, *et al. J Immunother Cancer* 2022; 8:e001240. doi: 10.1136/jitc-2020-001240

For subjects who withdraw from the study due to unacceptable toxicity without radiographic disease progression observed, the imaging examination should be performed at the same frequency, i.e., once every 8 weeks, until disease progression or start of other anti-tumor treatments. The radiographic evidence of PD of these subjects must be obtained whenever possible.

3.4 Detection of Plasma Tumor Burden and Gene Mutation Changes

At each imaging examination (± 2 days), blood samples are collected for detection of plasma tumor DNA and mutation.

3.5 Follow-Up within 28 Days after Discontinuation or before the Start of New Anti-tumor Treatment

Safety evaluations should be continued for all subjects within 28 days after the last administration or before the start of a new anti-tumor treatment. During the follow-up visit on D28 (± 3 days, except for statutory holidays) after the end of treatment, physical examination, ECOG PS score, hematology and blood biochemistry tests should be carried out, and AEs, concomitant medication and concomitant treatment should be evaluated.

3.6 Follow-Up of AEs

AEs that have not recovered after the discontinuation of SHR-1210 should be followed and finally assessed.AE follow-up is due on Day 28 after the last administration or the start of a new anti-tumor treatment, whichever occurs first.

3.7 Survival Follow-Up

After the 28-day safety follow-up, the subject, his/her family members or local physicians should be interviewed by telephone once a month to collect survival (date and cause of death) and post-treatment information (including received treatments) until the death endpoint, loss to follow-up, or study termination. Each survival follow-up should be documented in the follow-up form in detail.

4. RANDOMIZATION AND BLINDING

This is a single-arm, open-label, exploratory study and thus randomization and blinding are not required.

5. SELECTION AND WITHDRAWAL OF SUBJECTS

5.1 Inclusion Criteria

1. Pathologically confirmed biliary tract cancer, including intrahepatic cholangiocarcinoma, extrahepatic cholangiocarcinoma, and gallbladder cancer.
2. Males or females aged 18-75 years old;
3. Expected survival ≥ 3 months;
4. ECOG PS score of 0-1;
5. Have at least 1 measurable lesion as per RECIST 1.1 criteria.
6. No previous treatment with oxaliplatin, gemcitabine, or PD-1/PD-L1 monoclonal antibodies.
7. Patients who have previously received tegafur or capecitabine as postoperative adjuvant chemotherapy or first-line treatment may be enrolled.
8. Able to provide enough tissue samples for PD-L1 immunohistochemical testing and second-generation sequencing;
9. Major organs function well, meeting the following criteria for the relevant examination indicators within 14 days before enrollment:
   a) Hematology:
      i. Hemoglobin ≥ 90 g/L (no blood transfusion within 14 days);
      ii. Neutrophil count > 1.5×10^9/L;
      iii. Platelet count ≥ 100×10^9/L;
   b) Biochemistry:
      i. Total bilirubin ≤ 1.5 × ULN (upper limit of normal);
      ii. Blood alanine aminotransferase (ALT) or blood aspartate aminotransferase (AST) ≤ 2.5 × ULN; for those with liver metastases, ALT or AST ≤ 5 × ULN;
      iii. Endogenous creatinine clearance ≥ 60 mL/min (Cockcroft-Gault formula);
   c) Cardiac color Doppler: left ventricular ejection fraction (LVEF) ≥ 50%.
10. Signing informed consent form;
11. Subjects have good compliance and family members agree to corporate with the survival follow-up;
12. Female subjects: Have undergone surgical sterilization or be post-menopausal, or willing to take a medically approved contraceptive measure during and for 6 months after the study treatment; have a negative serum or urine pregnancy test within 7 days prior to enrollment, and not be breastfeeding.Male subjects: Have undergone surgical sterilization, or willing to take a medically approved contraceptive measure during and for 6 months after the study treatment;

5.2 Exclusion Criteria

1. Have other previous or concomitant malignant tumors, excluding cured basal cell carcinoma and cervical carcinoma in situ; patients with other tumors such as concomitant small gastric stromal tumor that do not affect the patient's life in the short term may also be excluded.
2. Participation in other drug clinical trials within the last 4 weeks;
3. With dysphagia, chronic diarrhea, intestinal obstruction or other diseases affecting the nutritional status of patients;
4. Have a history of bleeding, and any Grade ≥ 3 bleeding as per CTCAE 4.0 within 4 weeks before screening;
5. Patients with known CNS metastasis or a history of CNS metastasis before screening. For patients with clinically suspected CNS metastasis, CT or MRI examinations must be performed within 28 days before enrollment to rule out CNS metastasis;
6. Patients with a history of unstable angina; newly diagnosed with angina within 3 months before screening or myocardial infarction within 6 months before screening; arrhythmia (including QTcF: ≥ 450 ms in males, ≥ 470 ms in females) that requires long-term use of antiarrhythmic drugs and New York Heart Association Class ≥ II cardiac insufficiency;
7. Urine protein ≥ ++ or 24-h urine protein > 1.0 g as indicated by urinalysis;
8. Have used immune-targeted drugs;
9. Have undergone liver transplant or other organ transplant;
10. Patients with infectious pneumonia, non-infectious pneumonia, interstitial pneumonia, or other diseases requiring corticosteroids;
11. Have a history of chronic autoimmune diseases, such as systemic lupus erythematosus;
12. Have a history of ulcerative colitis, Crohn's disease or other inflammatory bowel diseases, and a history of chronic diarrheal diseases such as irritable bowel syndrome;
13. Have a history of sarcoidosis or tuberculosis;
14. Patients with history of active hepatitis B and C, and HIV infection; patients with hepatitis B virus DNA controlled at < 2000 IU/mL may be enrolled;
15. Patients with high sensitivity to human or murine monoclonal antibodies;
16. Have a history of psychotropic substance abuse that unable to quit or have mental disorders;
17. Have clinical symptoms, pleural effusion or peritoneal effusion that require clinical intervention;
18. Have a history of immunodeficiency or other acquired or congenital immunodeficiencies;
19. Presence of accompanying diseases that may pose serious risks to the safety of the patient or may affect the patient's ability to complete the study as judged by the investigator;

5.3 Study Withdrawal Criteria

The study treatment will be discontinued in any of the following cases:

1. Subject withdraws informed consent and requests to withdraw from the study;
2. Imaging evaluations show disease progression, unless the subject meets the criteria for continuation of treatment beyond progression (see Section 7.2.5 for details);
3. Continuing participation in the study is not in the best interests of the subject due to clinical adverse reactions, laboratory abnormalities, or concurrent diseases, as assessed by the investigator;
4. Subject is required to withdraw for other investigator-assessed causes. For example, the subject can no longer express voluntary consent due to incarceration or quarantine;

5.4 Termination Criteria

This study may be terminated under the conditions below:

This study may be terminated or suspended if there are valid reasons to do so. If this study is terminated early or suspended, the principal investigator must immediately notify the ethics committee and provide relevant reasons.

The termination criteria of this study include (but are not limited to):

- Discovery of unexpected, significant, or unacceptable risks to the subjects;
- Major errors in the protocol are found during the implementation of the trial;
- Due to reasons such as serious delay in the enrollment of subjects or frequent protocol deviations that make it extremely difficult to complete the trial.

6. STUDY DRUGS

6.1 Information

Investigational Drug: SHR-1210 for injection

Manufacturer: Shanghai Hengrui Pharmaceutical Co., Ltd.

Dosage form: lyophilized powder

Specification: 200 mg (proposed) in 20-mL vials. Batch number: ××××, Preparation date: ××××;

Route of administration: intravenous injection;

Shelf life: 2 years (tentative) from the date of manufacture.

Storage conditions: sealed, away from light, stored at 2-8 °C in medical refrigerator. Do not freeze.

Supplier: Shanghai Hengrui Pharmaceutical Co., Ltd., for patients to use for free.

Chemotherapy: Gemcitabine Hydrochloride Injection (Zefei)

Manufacturer: Jiangsu Hansoh Pharmaceutical Group Co., Ltd.

Dosage form: powder for injection

Specification: 1.0 g and 0.2 g

Route of administration: intravenous injection.

Shelf life:

Storage conditions: sealed and stored in a dry place.

Source: Routine medicine in the pharmacy of Jiangsu Province Hospital, for patients to buy and use normally.

Chemotherapy: Oxaliplatin Injection (AiHeng)

Manufacturer: Jiangsu Hansoh Pharmaceutical Group Co., Ltd.

Dosage form: powder for injection

Specification: 50 mg

Route of administration: intravenous injection

Chen X, *et al. J Immunother Cancer* 2022; 8:e001240. doi: 10.1136/jitc-2020-001240

Shelf life:

Storage conditions: sealed and stored below 25 °C.

Source: Routine medicine in the pharmacy of Jiangsu Province Hospital, for patients to buy and use normally.

6.2 Labeling/Packaging

The drug label is shown in Appendix 7. Specific drug packaging specifications and quantity: 200 mg/vial.

6.3 Drug Dispensation

After eligible patients are enrolled, drug administrators dispense corresponding drugs to each subject according to the order of the enrollment time.

The management, dispensing and return of the study drugs are in the charge of designated staff. The investigator must ensure that all the study drugs are only used for the subjects participating in this clinical trial. The dosage and administration should follow the trial protocol. The remaining drugs should be returned to the Hengrui. The study drugs should not be transferred to any non-clinical trial participant.

The study drugs are stored in a medical refrigerator at 2-8 °C. The drug receipt forms must be signed by two people during drug dispensing and in duplicate, one copy for Jiangsu Province Hospital and one copy for Hengrui. Remaining drugs and empty boxes are retrieved at the end of the study and a retrieval form will also be signed by both parties. The dispensing and return of every drug should be immediately documented on designated forms.

The monitor is responsible for monitoring the supply, usage and storage of the study drugs, and the management of remaining drugs.

6.4 Drug Storage and Management

The study drugs are stored, dispensed, and recovered by the Clinical Research Lab of the Oncology Department of Jiangsu Province Hospital. The study drugs should be sealed, kept away from light, and stored at 2-8 °C in medical refrigerator. Do not freeze.

SHR-1210 is not permitted to be used except for this study.

6.5 Disposal of Remaining Drugs

The investigator should record the date and dose of administration of each subject. The total dose of study drug assigned to each subject is 110% of the designed dose. Remaining study drugs should be returned to Hengrui after the end of each cycle.

6.6 Drug Preparation and Method of Administration

Since this product does not contain preservatives, please perform aseptic operations when formulating the drug preparation.

- Each vial of lyophilized powder is quantitatively reconstituted in 5 mL of distilled water for injection. During the operation, the distilled water is slowly added into the vial along the vial wall. Please do not directly drop the distilled water onto the surface of the lyophilized powder (the post-reconstitution concentration is 40 mg/mL)
- Do not violently shake the vial during reconstitution. Instead, reconstitute the powder in a gentle way with slow vortex. After reconstitution, allow the vial to stand for 6 min to allow the foam to disappear.
- Visually observe the solution after reconstitution to see whether there are particles and discoloration. After reconstitution, the liquid should be colorless or slightly yellowish. There may be a small number of light-gray small particles falling into the vial due to the puncture of vial stopper by the needle. However, these particles will be filtered by the 0.2 μM filter attached to the infusion set and hence will not affect the subsequent use of the drug solution. If there are other particles other than the ones mentioned above, please do not continue to use this vial of drug.
- Draw a corresponding volume of the reconstituted solution from the vial and dilute it in 100 mL of 5% glucose for injection. Avoid generating a large number of air bubbles during the dilution process. After dilution, slowly invert the infusion bag several times to mix well. Maintain the final concentration at between 0.5 mg/mL and 10 mg/mL.
- Within 2 h after the dilution is completed, use an infusion set equipped with an in-line filter (0.2 μM) to finish intravenous injection via a medical infusion pump. After the drug infusion is completed, use 20 mL of 5% glucose for injection to flush the drug remaining in the infusion line into the body. Do not use this infusion line to administer other drugs. The infusion should be completed in more than 30 min.

6.7 Concomitant Medication/Treatment

All treatments and medications used concomitantly within 1 month prior to informed consent and during the study are documented in the eCRF in strict accordance with the GCP regulations. Subjects should be closely monitored if adverse reactions occur, and active symptomatic treatment should be given if necessary. The drugs used should be documented and described in the eCRFs.

6.7.1 Drugs or treatment prohibited during the study

NMPA-approved Chinese medicine preparations for liver cancer (including Kanglaite injection, Aidi injection, and sodium cantharidinate) and immunomodulators (including but not limited to, interferon, interleukin-2, Zadaxin) are prohibited during the course of the study.

Local treatment for liver lesions and target lesions are not permitted. Systemic anti-tumor treatments such as chemotherapy, molecular targeted therapy, hormone therapy, immunotherapy, Chinese medicine (described above), and radiotherapy are not permitted.

Concomitant immunosuppressive therapy is not permitted (except when treating treatment-related adverse events).

Study drugs for cancer treatment

6.7.2 Medications and treatments that may be used concomitantly during the study

1. Anti-viral therapy

Subjects with HBV or HCV infections are required to receive anti-viral therapy according to local standard practices. Recommendations for antiviral therapy are as follows:

Subjects with HBV infection, such as ones who are HBV-DNA positive, who have started anti-viral therapy before enrolled into the study and with good control of viral load (HBV-DNA < 2000 IU/mL), should continue with the original anti-viral treatment. Those with poor viral load control need to switch to entecavir and can only be enrolled when the HBV-DNA is < 2000 IU/mL. HBV-infected subjects who are newly diagnosed at screening should start entecavir treatment immediately, and they can only be enrolled after HBV-DNA is < 2000 IU/mL.

Subjects with HCV infection, such as ones who are HCV-RNA positive, must receive anti-viral therapy according to standard practices.

2. Corticosteroids

Steroids that are permitted include local administrations of steroids, such as topical, ocular, nasal, intra-articular, and inhalation; cortisol for adrenal replacement therapies; cortisol for the treatment of adverse reactions; and temporary use for the prevention and treatment of allergic reactions (preventing allergic reactions to contrast agents, or treating other allergic reactions).

3. Vaccines

Vaccines that are used to prevent infectious diseases, such as pneumonia and influenza vaccines, are permitted. Any other vaccines should be discussed with the principal investigator before administered.

Chen X, *et al. J Immunother Cancer* 2022; 8:e001240. doi: 10.1136/jitc-2020-001240

4. Other systemic treatment

Subjects should be given optimal supportive care during the treatment. Existing hormone replacement therapies are permitted. Bisphosphonate treatment for bone metastasis is permitted.

5. Palliative local treatment

Subjects are permitted to receive palliative treatment of local lesions with clear symptoms, such as painful bone lesions. Local radiotherapy or surgery can be considered but must meet the following conditions: 1. The investigator must assess whether there is disease progression in subjects who require local treatment due to symptom exacerbations during the study; 2. Subjects with disease progression have to meet the criteria for continuation of treatment beyond progression (see Section 7.2.5); 3. The locally treated lesions should not be the target lesions.

The content of palliative therapy should be recorded in the CRF and medical record in details, including treatment period, site, method and dosage, as well as adverse reactions.

6.7.3 Recommended symptomatic treatment for common adverse reactions

6.7.3.1 Infusion reaction

During the course of this study, the investigator should pay close attention to potential infusion and/or allergic reactions, especially acute immune-mediated adverse reactions (including cytokine storms).

In general, no administration of prophylactics before the infusion of SHR-1210 is required. Based on published relevant information, an allergic reaction/event is most likely to occur within 24 h after infusion. If an allergic reaction/event occurs, the infusion should be slowed or interrupted according to the situation, and a supportive treatment should be given. In addition, prophylactics should be given before further administration. Possible allergic reactions include fever, chills, shiver, headache, rash, pruritus, joint pain, hypotension/hypertension, or bronchospasm. Any Grade 3 or 4 infusion reactions must be reported to the sponsor within 24 hours, and should be reported as SAEs if the criteria for SAE are met.

Management of allergic reactions should be based on the medical practice and guidelines of the trial site. The treatment recommendations for infusion reactions are shown below for reference.

CTCAE Grade	Clinical Symptoms	Management	SHR-1210 Treatment
Grade 1 (mild)	Mild and transient reaction; infusion interruption not indicated; intervention not indicated	- Bedside observation and close monitoring until recovery. (Premedication is recommended for subsequent infusion: diphenhydramine 50 mg or equivalent, and/or acetaminophen 325-1000 mg, at least 30 minutes prior to SHR-1210 administration)	Continuation
Grade 2 (moderate)	Infusion interruption indicated, give symptomatic treatment promptly (such as antihistamines, NSAIDs, sedatives, and IV fluids, etc.)	- Intravenous administration of normal saline: diphenhydramine 50 mg (IV) or equivalent and/or acetaminophen 325-1000 mg;<br>Bedside observation and close monitoring until recovery. ;<br>Corticosteroids may be considered;<br>The amount of study drug infused should be recorded in the CRF;<br>Premedication is recommended for subsequent infusion: diphenhydramine 50 mg or equivalent, and/or acetaminophen 325-1000 mg, at least 30 minutes prior to SHR-1210 administration. Use corticosteroids (equivalent to 25 mg of hydrocortisone) when necessary.	Interruption.<br>**Re-administer** at 50% of the initial rate after symptoms resolve. Restore the original infusion rate (100%) if no complications occur within 30 minutes. Closely monitor. Discontinue administration if symptoms recur.
Grade ≥ 3 (severe)	Grade 3: Prolonged (e.g., not rapidly responsive to symptomatic medication and/or brief interruption of infusion), or recurrence of symptoms following improvement.<br>Grade 4: Life-threatening	Immediately discontinue SHR-1210;<br>Intravenous administer normal saline.<br>• Bronchodilators are recommended. 0.2-1 mg of subcutaneous 1:1000 adrenaline injection or slow intravenous injection of 1:10000 adrenaline solution 0.1-0.25 mg, and/or intravenous diphenhydramine 50 mg plus methylprednisolone 100 mg or equivalent if necessary;<br>• Based on the guidelines for anaphylaxis of the study site;<br>Bedside observation and close monitoring until recovery.	Permanent discontinuation

6.7.3.2 Management principles for immune-related adverse reactions

In principle, interruption of SHR-1210 is preferred based on the severity of the adverse reaction. SHR-1210 treatment can be considered to resume when AE returns to Grade ≤ 1. SHR-1210 treatment should be permanently discontinued if severe (Grade 3) or life-threatening (Grade 4) adverse reactions occur.

The treatment of immune-related adverse reactions should be based on the medical practice and guidelines of the study site. The treatment recommendations for immune-related adverse reactions are as follows (see Table 4) for reference.See Appendix X for the management procedures for common immune-related adverse reactions

Table 4. Treatment recommendations for immune-related adverse reactions.

CTCAE Grade	Clinical Management*	SHR-1210 Treatment
Grade 1 (mild)	- Close observation, especially for diarrhea<br>• Supportive treatment	Continuation
Grade 2 (moderate)	- Close monitoring<br>• Supportive treatment<br>• Local application of steroids, such as for dermatitis/colitis, etc.<br>Symptoms persist for ≥ 7 days, start 1 mg/kg of prednisone or equivalent<br>Symptoms are aggravated, give intravenous or oral administration of 1 mg/kg prednisone	Interrupt, resume when recovers to ≤ Grade 1
≥ Grade 3 (Severe)*	-2 mg/kg of prednisone or equivalent	Permanent discontinuation

* The treatment may not be interrupted for local skin and Grade 3 endocrine disorders, because they can often heal (skin) or be treated using an alternative treatment (endocrine) (Weber, Jeffrey S. MD, PhD, et al., "Toxicities of immunotherapy for the practitioner," J Clin Oncol, April 2015)

7. DOSE MODIFICATION AND SAFETY MANAGEMENT

7.1 Dose Level and Use of Drugs

3 mg/kg, I.V, q2w, by an intravenous infusion over 30 minutes, until disease progression, unacceptable toxicities, withdrawal of informed consent or the end of study (whichever occurs first).

The subjects must be administered at an interval of no less than 12 days.

7.2 Dose Modification

7.2.1 Criteria for dose modification

Dose modification for study drugs are not permitted.

7.2.2 Criteria for dose delay

Administration should be delayed when any of the following occurs:

- Any Grade ≥ 2 treatment-related non-cutaneous AEs, except for Grade 2 treatment-related fatigue or laboratory abnormalities;
- Any Grade 3 treatment-related cutaneous AEs;
- Any Grade 3 treatment-related laboratory abnormalities, except for Grade 3 abnormalities in pancreatic enzyme or lipase not related to clinical manifestations of pancreatitis;
- Administration should be delayed under the AST or ALT conditions below:
  - If the baseline AST/ALT of a subject is within the normal range and a Grade ≥ 2 drug-related toxicity occurs, the administration should be delayed;
  - If the baseline AST/ALT of a subject is within a Grade 1 increase and a Grade ≥ 3 drug-related toxicity occurs, the administration should be delayed;
  - If the baseline AST/ALT of a subject is within a Grade 2 increase and AST/ALT increases by two-fold or AST/ALT exceeds eight-fold of upper limit normal (whichever is lower), the administration should be delayed;
- Any AEs, laboratory abnormalities, or accompanying diseases requiring that the administration be delayed as judged by the investigator

Subjects who need delay of administration should be re-examined and monitored weekly. Monitoring frequency should be increased when clinically indicated. It is recommended to monitor once every three days until AST/ALT starts to decrease after reaching its maximum. Administration may be resumed when criteria for resuming administration are met (see Section 7.2.3).

Tumor evaluation in all subjects will continue as required by the protocol, regardless of whether the administration is delayed.

7.2.3 Criteria for resuming administration

Study treatment may be resumed when drug-related AEs recover to Grade ≤ 1 or baseline status, except for:

- Subjects with Grade 2 fatigue that is not recovered may resume study treatment;
- Subjects with treatment-related cutaneous AEs that never reach Grade 3 may resume study treatment when the cutaneous AE is still Grade 2;
- Subjects with Grade 1 increase in AST/ALT or total bilirubin at baseline who delay administration due to causes other than treatment-related hepatic AEs may resume study treatment in the presence of Grade 2 increase in AST/ALT or total bilirubin;
- Subjects who delay administration due to treatment-related increase in AST/ALT or total bilirubin may resume study treatment when these indicators recover to baseline CTCAE levels or normal and criteria for permanent treatment discontinuation are not met (see Section 7.2.4).
- Administration may be resumed after treatment-related pulmonary toxicity, diarrhea, or colitis returns to baseline.
- Administration may be resumed if treatment-related endocrine illnesses are fully controlled with hormone replacement at physiological doses.

Dose delays up to 4 weeks from the previous dose are allowed. If, after a 4-week delay, the subject still does not meet criteria for resuming administration, study treatment should be discontinued permanently, except in conditions in Sections 7.2.4 and 7.2.5. See Appendix X for the detailed treatment measures for adverse reactions.

7.2.4 Criteria for permanent discontinuation

Study treatment must be permanently discontinued under the conditions below:

- Any Grade 2 treatment-related uveitis, eye pain, and blurred vision that are not responsive to local treatment and have not recovered to Grade 1; or the aforementioned AEs that require systemic treatment.
- Any Grade 3 treatment-related non-cutaneous AEs persisting > 7 days, except for:
  - Study treatment must be discontinued if any Grade 3 treatment-related uveitis, pneumonia, bronchospasm, hypersensitivity, or infusion reaction occur;

  - Study treatment may not be discontinued if Grade 3 treatment-related endocrine illnesses are fully controlled with hormone replacement therapy at physiological doses;
  - Study treatment may not be discontinued if Grade 3 treatment-related laboratory abnormalities occur, but must be discontinued if Grade 3 thrombocytopenia persists > 7 days or is related to bleeding.
- Hepatotoxicity that meets the criteria below:
  - AST/ALT > 10 × ULN for > 2 weeks;
  - AST/ALT > 15 × ULN;
  - TBIL > 8 × ULN for subjects with increased TBIL at baseline, and TBIL > 5 × ULN for subjects with normal TBIL at baseline;
  - AST/ALT > 3 × ULN and TBIL > 5 × ULN for subjects with normal TBIL at baseline, and AST/ALT > 3 × ULN and TBIL > 8 × ULN for subjects with increased TBIL at baseline;
- Any Grade 4 treatment-related AEs or laboratory abnormalities, except for:
  - Grade 4 neutropenia for < 7 days;
  - Grade 4 lymphopenia or leukopenia;
  - Solitary Grade 4 pancreatic enzymes or lipases elevated without pancreatitis symptoms or clinical manifestations;
  - Isolated Grade 4 electrolyte imbalances/abnormalities that are not accompanied by clinical sequelae and can be corrected with supplements/appropriate treatment within 72 hours of their occurrence;
  - Study treatment may not be discontinued if Grade 4 treatment-related endocrine illnesses are fully controlled with hormone replacement therapy at physiological doses.

- Study treatment must be discontinued if dose delay of > 4 weeks is needed, except for:
  - Dose delays of > 4 weeks due to dose tapering of corticosteroids that are used for treatment-related AEs are permitted. Tumor evaluations should be continued as required by the protocol during dose delay. Safety follow-ups and laboratory tests should also be performed at the original frequency or more frequently when clinically indicated.
  - Dose delays of > 4 weeks due to reasons unrelated to study drugs. Tumor evaluations should be continued as required by the protocol during dose delay. Safety follow-ups and laboratory tests should also be performed at the original frequency or more frequently when clinically indicated.
- Any AEs, laboratory abnormalities, or accompanying diseases that render the subject significant risks from continued treatment as judged by the investigator.
- Disease progression assessed by the investigator as per RECIST 1.1 criteria (unless the subject meets the criteria in Section 7.2.5)

Tumor evaluations should be conducted for all subjects discontinuing treatment, as required by the protocol.

7.2.5 Criteria for continuing treatment beyond progression

Some subjects receiving immunotherapy can still benefit clinically after radiographic progression. Study treatment may be continued after disease progression defined per RECIST 1.1 for subjects who meet the criteria below:

- The investigator deems that it is in the best interest of the subject to continue treatment, and subject is not required to start other anti-tumor treatment immediately;
- The subject is able to tolerate continued study treatment;
- No significant deterioration in subject's performance status, and no significant worsening of cancer-related symptoms;
- Subjects must re-sign the informed consent form prior to continuing treatment, in which potential risks, discomforts, and other treatment options shall be included;
- Continued treatment must be reviewed and approved by the PI

The assessment of clinical benefit must consider whether the subject has clinical exacerbations and whether the subject can benefit from continuing treatment. Decisions of continuing treatment after progression as judged by the investigator should be discussed with the PI and documented in the study records.

If it is decided that the subject will continue the study treatment after progression, the subject should continue to be treated, evaluated and followed up according to the protocol requirements.

Subject should withdraw from study treatment if further progression is observed at the next assessment. The initial date of investigator-assessed progression should be used for all statistics analyses involving progression, regardless of whether the subject continues the study treatment beyond progression.

If the subject discontinues treatment due to deterioration of the general condition without objective evidence for disease progression, the progression will be reported as "general deterioration". More objective evidences (such as imaging confirmation) of progression of these subjects should be obtained after treatment discontinuation.

7.3 Safety Management for Immuno-Oncological Medications

7.3.1 Safety management procedures for immuno-oncological medications

Adverse reactions caused by immuno-oncology (I-O) drugs are different from those of other anti-cancer drugs, especially in terms of severity and duration. SHR-1210 is one such drug, and therefore, early identification and management of adverse events is required to reduce the incidence of severe toxicities. The safety management procedures of similar approved drugs provide references to assist the investigator in assessing and dealing with adverse events involving the following systems:

- GI tract
- Kidney
- Lung
- Liver
- Endocrine
- Skin
- Nervous system

7.3.2 Management procedures for hepatic AEs

In this study, hepatic AEs are treated with the procedures below:

- Criteria for dose delays due to hepatic AEs are listed in Appendix X. Corticosteroids, methylprednisolone 0.5-2 mg/kg/day, or equivalent should be given if the AST/ALT level does not improve or even aggravates following a dose delay of 3-5 days.
- Corticosteroids, methylprednisolone 0.5-2 mg/kg/day or equivalent should be given immediately if AST/ALT > 8 × ULN.
- The sponsor shall be informed of the use of corticosteroids within 24 hours of its start. Consultation with the gastroenterology department is also recommended.
- The sponsor should be consulted for the use of other immunosuppressants such as mycophenolate 1 g BID if the AST/ALT level does not improve or even exacerbates following treatment with corticosteroids of 3-5 days.
- Dose tapering may start once AST/ALT decreases by 1 grade per CTCAE, with the dose reduction persisting no less than 1 month.

Study treatment may be resumed when AST/ALT recovers to baseline level, unless the criteria for permanent discontinuation are met.

8. STUDY PROCEDURES

8.1 Screening (from signing informed consent form to confirmed enrollment)

Subjects must sign the informed consent form before undergoing any screening procedures.

Unless otherwise stated, the following screening procedures should be completed within 28 days prior to randomization.

- Patients sign the informed consent form.
- Collection of medical history, including past treatment history, current medical history, drug allergies, and concurrent diseases.
- Collect 15-20 slides of histopathological specimens (Detection of PD-L1, CD8+ TILs, and TMB).
- Physical examination, height, weight, and vital signs (temperature, blood pressure, heart rate, and respiratory rate).
- Chest x-ray, not required if the subject have undergone chest CT.

- CT or MRI of the chest, abdomen, pelvis and other relevant lesions.
- Brain CT/MRI, required for clinically suspected metastases to the central nervous system only.
- Bone scan, required for clinically suspected bone metastasis only.

The following information should be collected within 14 days before confirmed enrollment:

- ECOG PS score;
- Hematology, urinalysis, and routine stool test.
- Hepatic and renal function (total bilirubin, direct bilirubin, indirect bilirubin, ALT, AST, AKP, r-GT, LDH, total protein, albumin, blood urea nitrogen, creatinine, and uric acid).
- Electrolytes (potassium, sodium, chlorine, calcium, magnesium);
- Coagulation function (PT or INR).
- Serum pregnancy test (for women of childbearing potential).
- Detection of hepatitis B and C markers, HBV DNA, and HCV-RNA.
- Detection of serum AFP, CEA, CA199.
- Echocardiography (mainly used for LVEF).
- 12-Lead ECG (the QTc interval must be indicated).
- Confirmation of the assigned study number and corresponding drug number after completing all screening evaluations.

8.2 Treatment Period

Each cycle during the treatment period is defined as 2 weeks. As per RECIST v1.1, the first tumor imaging evaluation is performed at Week 8 (± 7 days) after the first dose, and once every 8 weeks (± 7 days) thereafter regardless of drug administration, until disease progression or treatment discontinuation (whichever happens last).

See Schedule of Activities for detailed examinations.

8.3 Follow-Ups

8.3.1 Follow-up of AEs

AEs that have not resolved after the discontinuation of SHR-1210 should be followed and finally assessed. AEs should be followed until Day 28 after dose discontinuation.

8.3.2 Survival follow-up

After the 28-day safety follow-up, the subject, his/her family members or local physicians should be interviewed by telephone once a month to collect survival (date and cause of death) and post-treatment information (including received treatments) until the death endpoint, loss to follow-up, or study termination by Hengrui. Each survival follow-up should be documented in the follow-up form in detail.

9. EFFICACY EVALUATION

9.1 Primary Endpoints and Observations

1) 6-month PFS%: Refers to the proportion of evaluable patients who survive from the start of the trial to 6 months.
2) Drug safety: vital signs, laboratory measurements, AEs, SAEs, treatment-related AEs and SAEs, as per NCI-CTCAE V4.0 criteria.

9.2 Secondary Endpoints and Observations

1) Objective response rate (ORR)

Refer to using the RECIST version 1.1 criteria to evaluate the objective tumor response, including the CR and PR cases.

Definition of evaluable subjects: All subjects who have received at least one SHR-1210 treatment and have undergone at least one tumor evaluation after the start of study treatment. CR or PR must be confirmed at least 4 weeks (28 days) after the first assessment.

2) Duration of response (DoR)

Refer to the time from imaging confirmed CR or PR to any documented radiographic disease progression.

3) Disease control rate (DCR)
4) 12-month survival
5) Overall survival (OS)

Refers to the time from the date of enrollment to death of any cause.

Chen X, et al. J Immunother Cancer 2022; 8:e001240. doi: 10.1136/jitc-2020-001240

9.3 Exploratory Endpoints and Observations

1) Relationship between PD-L1 expression and DCR and 6-month PFS%

PD-L1 expression in patients is detected using Ventana SP263 antibodies, including TPS and IPS.

The relationship between PD-L1 expression and DCR and 6-month PFS% is analyzed.

2) Measurements for CD8+ TILs and other immune microenvironment

The relationships of the expression in CD8+ TILs and other immune microenvironment with DCR and 6-month PFS% are analyzed.

3) TMB

The tumor mutation burden is detected by Geneseeq Technology Inc.

Plasma tumor mutational burden and changes of ctDNA amount before and two months after treatment.

10. SAFETY EVALUATION

10.1 Adverse Event (AE)

10.1.1 Definition of AE

An AE is defined as any untoward medical condition that occurs after the signing of the informed consent form until 28 days after the last study dose, and which does not necessarily have a causal relationship with the study drug AEs include the followings:

1) Worsening of pre-existing (prior to entering clinical trial) medical conditions/diseases (including worsening symptoms, signs, or laboratory abnormalities);
2) Any new AE: Any new adverse medical conditions (including symptoms, signs, and newly diagnosed diseases);
3) Clinically significant abnormal laboratory findings.

All AEs should be documented in detail by the investigators, including: the name of the AE and description of all relevant symptoms, onset time, severity, causality assessment, duration, measures taken, as well as final results and outcomes.

10.1.2 AE severity grading criteria

The severity of AE is determined using NCI-CTCAE 4.03. Refer to the following criteria for AEs not listed in NCI-CTCAE 4.03:

Grade	Clinical Description of Severity
1	Mild, asymptomatic or mild symptoms; clinical or diagnostic observations only; intervention not indicated
2	Moderate; minimal, local, or non-invasive interventions required; limited age-appropriate instrumental activities of daily living (ADL), e.g., cooking, shopping, using the telephone, counting money, etc.
3	Severe or medically significant symptoms but not immediately life-threatening; hospitalization or prolongation of hospitalization indicated; disabling; limiting self-care ADL. Self-care ADL: refer to bathing, dressing and undressing, feeding self, using the toilet, taking medications, and not bedridden
4	Life-threatening consequences; urgent intervention indicated
5	Death due to AEs

10.1.3 Causality assessment

AEs include all unexpected clinical manifestations. All such events occurring after the signing of the ICF must be reported as AEs regardless of whether they are related to the study drug, whether the subject is allocated to the treatment group, and whether the subject has received the drug. Any discomforts complained by the subject or abnormal changes in laboratory measurements during the treatment should be truthfully documented, noting the severity of AEs, duration, measures taken, and outcome. The investigator should comprehensively determine the relationship between the AEs and the study drug, and provide a causality assessment using the following five categories "definitely related, possibly related, unlikely related, definitely unrelated, and indeterminable". Those "definitely related", "possibly related", and "indeterminable" are all listed as adverse drug reactions. The criteria are as follows Table 5:

Table 5. Evaluation of causality between AEs and study drug.

Classification	Criteria
Definitely Related	The AE occurs in a plausible time relationship to drug administration. The event is a recognized pharmacological phenomenon of the suspected drug. Or, the event resolves with drug discontinuation and recurs with drug readministration.
Possibly Related	The AE occurs in a plausible time relationship to drug administration. The event is not a recognized pharmacological phenomenon of the investigational product. It can also be explained by patient's clinical status or other treatments.
Unlikely Related	The AE does not occur in a plausible time relationship to drug administration. The event is not a recognized pharmacological phenomenon of the investigational product. It can also be explained by patient's clinical status or other treatments.
Definitely Unrelated	The AE does not occur in a plausible time relationship to drug administration. The event is not a recognized pharmacological phenomenon of the investigational product. It can also be explained by patient's clinical status or other treatments. The event resolves when patient's clinical status improves or other treatments are discontinued. The event recurs upon restarting other treatment.
Indeterminable	The time relationship between the AE and drug administration is unclear. The event is similar to a recognized pharmacological phenomenon of the investigational product. It can also be explained by combined medications.

Chen X, *et al. J Immunother Cancer* 2022; 8:e001240. doi: 10.1136/jitc-2020-001240

10.2 Serious Adverse Event (SAE)

10.2.1 Definition of SAE

SAE refers to a medical occurrence during the clinical trial that results in hospitalization, prolonged hospitalization, disability, incapacity, life-threatening or death, or congenital malformation. The following unexpected medical events are included:

- Events resulting in death;
- Life-threatening events (defined as when the subject is at immediate risk of death at the time of the event);
- Events resulting in hospitalization or prolonged hospitalization;
- Events resulting in permanent or serious disability/incapacity/impairment of work ability;
- Congenital anomalies or birth defects;
- Other important medical events (defined as events that may jeopardize the subject or require interventions to prevent any of the above).

10.2.2 Disease progression

A PD is defined as the deterioration of the subject's conditions caused by the primary tumor that the investigational product is targeting, including radiological progressions and progressions in clinical symptoms and signs. New metastases relative to the primary tumor, or progression of the previous metastases, are recognized as PD. Life-threatening events, hospitalization or prolonged hospitalization, permanent or serious disability/incapacity/impairment of work ability, congenital anomalies or birth defects resulting from signs and symptoms of progressive disease should not be reported as SAEs on an expedited basis. Death caused by the symptoms and signs of PD is reported as an SAE on an expedited basis.

10.2.3 Other anti-tumor treatments

AEs should be recorded from the signing of the informed consent form until 30 days after the last study dose or the start of new anti-tumor treatment. AEs except death will not be followed and recorded any more. Deaths that occur within the SAE reporting period after study treatment is completed should be reported regardless of whether the subject has received other treatment.

Chen X, *et al. J Immunother Cancer* 2022; 8:e001240. doi: 10.1136/jitc-2020-001240

10.2.4 Hospitalization

AEs that lead to hospitalization or prolonged hospitalization during clinical trial should be considered SAEs. Any initial hospital admission by a medical facility meets this criterion.

Hospitalization does not include the following:

- Hospitalization at a rehabilitation institution
- Hospitalization at a sanatorium
- General emergency admission
- Day surgery (e.g., outpatient/same-day/ambulatory surgery)

Hospitalization or prolonged hospitalization unrelated to the worsening of an AE is not an SAE. For example:

- Hospitalization due to pre-existing disease without occurrence of new AEs or worsening of the pre-existing disease (e.g., in order to examine the persistent laboratory abnormalities that started before the study);
- Hospitalization for management reasons (e.g., annual routine physical examination);
- Hospitalization during the study as specified in the study protocol (e.g., as required by the protocol);
- Elective hospitalization unrelated to worsening of AEs (e.g., elective surgery);
- Scheduled treatment or surgery that should be documented throughout the entire study protocol and/or subjects' individual baseline information;
- Hospitalization merely for the use of blood products.

Diagnostic or therapeutic invasive (e.g., surgery) and non-invasive procedures should not be reported as AEs. However, when a condition resulting in such procedures meet the definition of an AE, it should be reported as so. For example, acute appendicitis during the AE reporting period should be reported as an AE, and the resulting appendicectomy shall be recorded as the treatment of the AE.

10.2.5 SAE reporting

The reporting period for SAE begins with the signing of the informed consent form until 28 calendar days (inclusive) after the last study dose. In the event of an SAE, whether it is the first report or a follow-up report, the investigator must complete the "New Drug Clinical Trial Serious Adverse Event (SAE) Report Form" immediately, with a signature and date. It must be reported to the provincial, autonomous regional and municipal drug regulatory authorities, NMPA (via EMS), health administration (via fax to bureau of medical administration), and the ethics committee within 24 hours of knowing the event. Refer to Table 6 below for contact details.

SAEs that occur *28* days after the last study dose are generally not reported unless they are suspected to be related to the study drug.

The symptoms, severity, relationship with the study drug, time of occurrence, treatment duration, measures taken, time and method of follow-up, and outcome should be documented in details in the SAE report. If the investigator believes that an SAE is not related to the study drug but potentially related to the study conditions (such as the termination of past treatment, or comorbidities during the trial), their relationship should be explained in the description section of the SAE report form.

If the severity of an SAE or its relationship to the study drug changes, a follow-up report should be submitted immediately.

Table 6. SAE report contacts.

Unit	Department	Fax/Telephone/Address
Hospital	Ethics Committee	Telephone/Fax:
National Medical Products Administration	Division of Drug Research Supervision, Department of Drug and Cosmetics Registration	Address: Building 2, No. 26, Xuanwumen West Street, Xicheng District, Beijing<br>Postal Code: 100053<br>Tel.: 010-88330732<br>Fax: 010-88363228
Health Administration	Bureau of Medical Administration	Address: No. 38, North Lishi Road, Xicheng District, Beijing (100810)<br>Tel.: 010-68792201<br>Fax: 010-68792734 (preferred)
Food and Drug Administration of Provinces, Autonomous Regions and Municipalities	Refer to the reporting requirements of the drug administration department of each province, autonomous region, or municipality	

10.2.6 Follow-up of SAEs

All SAEs should be followed until disappeared, resolved to baseline level or Grade ≤ 1, or a stable state.

AEs should be followed-up until the end of the event, stable condition, reasonable explanation available, loss to follow-up, or death. Follow-up information should be readily available when requested by the sponsor.

If not otherwise required by the protocol, collection and follow-up of AEs/SAEs usually starts after the subject signs the informed consent form, with each AE/SAE followed during the study period. At the end of the study period, AEs/SAEs that occur after the last study dose should be collected and followed with reference to the table below:

Classification	Collection and Documentation	Follow-up
Unrelated AEs	*28* days after the end of treatment	*28* days after the end of treatment
Related AEs	3 months after the end of treatment	Until the event resolves, or returns to baseline or a stable status
Unrelated SAEs	*28* days after the end of treatment	*28* days after the end of treatment
Related SAEs	No requirement	Until the event resolves, or returns to baseline or a stable status

10.3 Pregnancy

During the study, if a female subject becomes pregnant, she must discontinue the study drug immediately. The investigator must report to the sponsor within 24 hours and fill out the Pregnancy Report/Follow-up Form for Hengrui Clinical Studies.

During the study, if the partner of a male subject becomes pregnant, the subject can continue in the study. The investigator must report to the sponsor within 24 hours and fill out the Pregnancy Report/Follow-up Form for Hengrui Clinical Studies.

The investigator should follow the pregnancy until 1 month after delivery, and report the results to the sponsor.

Pregnancy outcomes such as stillbirth, spontaneous abortion and fetal malformation are considered SAEs and need to be reported according to the time requirements for SAEs.

If the subject also experiences an SAE during the pregnancy, the NMPA SAE Report Form should also be filled out and reported according to the procedures of SAE reporting.

10.4 AEs of Special Interest

For AE of special interest specified in the trial protocol, the investigator must fill out the "Report of Adverse Event of Special Interest for Clinical Studies".

If an AE of special interest is also an SAE, the "NMPA Serious Adverse Event Report Form" must also be completed.

- Grade ≥ 3 infusion reaction
- Grade ≥ 2 diarrhea/colitis, uveitis, interstitial pneumonia
- Other Grade ≥ 3 immune-related AEs;
- Any possible hepatic enzyme abnormalities (ALT/AST > 3 × ULN accompanied by TBIL > 2 × ULN, and lack of other relevant causes)

11. DATA MANAGEMENT

11.1 Data Collection and Management

Data will be collected and managed using the electronic case report form (eCRF).

11.1.1 Data collection

This study will use eCRF to collect study data with an electronic data capture (EDC) system provided by Jiangsu Province Hospital. Access to EDC system will only be granted to the investigator and relevant personnel who have completed the training on EDC system. The PI or dedicated data entry person (CRC) should input data into the EDC system in accordance with the requirements of the visit procedures and the eCRF completion guide. The logic verification program in the system will verify the integrity and logic of the clinical trial data entered into the EDC system and generate an error message prompt for questionable data. The PI or CRC is permitted to modify or explain the problematic data.

11.1.2 Data management and quality control

To ensure authenticity and reliability and improve the quality of the clinical data, the CRA will monitor the integrity, consistency, and accuracy of the trial data in the database, and guide the study site staff to add or correct the data whenever necessary. The CRA or data manager will send electronic query forms to the PI or CRC for questionable data. The PI or CRC must respond to the queries and correct or explain the data in question. When necessary, queries may be raised multiple times until the issue is resolved. The medical director and data manager should perform consistency comparison of SAEs periodically.

Chen X, et al. J Immunother Cancer 2022; 8:e001240. doi: 10.1136/jitc-2020-001240

At the end of the study, the data manager and medical personnel will conduct a final quality control of all data in the database, summarize all protocol deviations and violations during the trial, and hold a data verification meeting. The database will be locked after the quality requirements are met. The data manager will export the data to the statistician for data analysis.

11.1.3 Data review and study site monitoring

The investigator must keep the source documents of each subject, including all medical records and visit records (outpatient or inpatient record), such as demographic indicators, medical information, lab results, ECGs, and result of other examinations and evaluations. All information on the eCRF must come from the source documents of the subject. The investigator must also keep the informed consent forms signed by the subjects.

The investigator must ensure all source documents are available for monitoring to ensure the consistency with the eCRF. No information related to subjects' identity in the source document will be disclosed.

12. DATA ANALYSIS AND STATISTICS

Detailed summaries and methods of statistical analyses for the data collected from the study will be included in the statistical analysis plan (SAP), which will be finalized and filed by the sponsor. The SAP should be revised accordingly if there are any changes to the study protocol that may have a significant impact on the SAP, as determined by the sponsor or principal investigator.

12.1 Determination of Sample Size

In previous clinical studies of gemcitabine combined with oxaliplatin as first-line treatment of advanced BTC, the median survival of patients was 3-5.5 months, and the 6-month PFS was 30-40%. Of these reports, two were from East Asian populations (Korea) with a 6-month PFS of approximately 30%. In the BINGO study, the 6-month PFS of the GEMOX group was approximately 40%. With reference to these studies, assuming a 6-month PFS of 40% in the first-line treatment of gemcitabine combined with oxaliplatin in patients with advanced BTC in China, the 6-month PFS in this study is expected to be increased to 60%; with one-sided test, alpha of 0.05, and power of 80%, a sample size of 35 subjects is required. Considering a drop-out rate (within 10%) in study visits, 38 subjects need to be enrolled.

12.2 Analysis Population

Full analysis set (FAS): The analysis set determined based on the intention-to-treat principle. All enrolled subjects who have received at least one study dose are included in this analysis set. The FAS is the primary set for efficacy analysis in this study.

Per-protocol set (PPS): A subset of the FAS. Subjects with protocol violations that are judged to have a significant effect on the efficacy will be excluded from this set. The list of subjects included into or excluded from the PPS should be reviewed and determined by the sponsor and the investigator before the database is locked.

Definition of evaluable subjects: All subjects who have received at least one SHR-1210 treatment and have undergone at least one tumor evaluation. Subjects must undergo efficacy confirmation for an efficacy of CR or PR

Safety set (SS): All enrolled subjects with at least one administration record will be included in the safety set.

PD-L1/CD8+ TILs/TMB analysis set: All enrolled subjects who have received at least one study dose and provided tumor biopsy samples constitute the PD-L1 analysis set for this study.

Plasma tumor molecular burden and gene mutation change analysis set: All subjects who have received study treatment, have at least one imaging evaluation, and have corresponding blood samples are included in the mutation analysis set.

12.3 Statistical Analysis

12.3.1 General analysis

Unless otherwise stated, continuous variables will be summarized by mean, standard deviation, median, maximum, and minimum; categorical variables will be summarized by frequencies and percentages; for time-event data, the survival rate and median survival will be estimated using the Kaplan-Meier method.

12.3.2 Efficacy analysis

Tumor evaluations will be conducted by the investigator as per RECIST 1.1. The evaluation data will be based on the following indicators: complete response (CR), partial response (PR), stable disease (SD), disease progression (PD), and not evaluable (NE). All efficacy analyses will be conducted based on the FAS.

The OS distribution will be estimated using the Kaplan-Meier (KM) method and the median OS and two-sided 95% CI will be calculated. In addition, the KM method will be used to estimate the survival probability at different time points (at Months 6/9/12), and to calculate the corresponding 95% CI.

The PFS distribution will be estimated using the Kaplan-Meier (KM) method and the median PFS and two-sided 95% CI will be calculated.

The objective response rate (ORR) and disease control rate (DCR) and their 95% CIs will be calculated using the Clopper-Pearson method.

12.3.3 Safety analysis

AEs are analyzed based on the safety set. Data analysis includes but is not limited to the incidence of adverse reactions in each group. Frequency and cases of adverse reactions is summarized by organ system, and the number of cases and percentages of AEs are calculated. Details are described in the Statistical Analysis Plan.

12.3.4 Quality of life analysis

ORTC QLQ-C30 and HCC-18 will be analyzed using descriptive statistics. The analysis set is FAS.

12.3.5 Exploratory analysis

The expression of PD-L1 and CD8+ TILs will be analyzed using descriptive statistics.

TMB, plasma tumor molecular burden, and gene mutation changes will be analyzed using descriptive statistics.

13. INFORMED CONSENT AND ETHICS

13.1 Informed Consent

The clinical investigator must fully inform the subjects that participation in the clinical trial is voluntary, and subjects have the right to withdraw from the trial at any stage without being discriminated against with their medical treatment and rights unaffected, and they can continue to receive other therapies. All subjects should be informed that the participation of the trial and their personal information will be kept confidential. The subjects should also be informed of the nature, objectives, expected potential benefits, and possible risks and inconvenience of the clinical trial, other alternative treatment options, and rights and obligations of the subjects in accordance with the "Declaration of Helsinki". Subjects are given sufficient time to consider whether to participate in the trial and sign the informed consent form.

13.2 Ethics

This study protocol must first be reviewed and approved in writing by the Ethics Committee of the Hospital before being implemented. The study protocol, protocol revisions, ICF, and other relevant documents such as recruitment advertisements should be submitted to the ethics committee. This clinical trial must be conducted in accordance with the Declaration of Helsinki, NMPA's GCP, and applicable laws and regulations. Before the trial is initiated, approval must be obtained from the ethics committee of the hospital.

Chen X, *et al. J Immunother Cancer* 2022; 8:e001240. doi: 10.1136/jitc-2020-001240

The investigator shall not modify this study protocol at will. The investigator can modify or deviate from the study protocol before obtaining an approval from the IRB/IEC only when in purpose of eliminating direct and immediate harm to the subject. Besides, the deviation or change and the corresponding reason, and the recommended protocol modification should be submitted to the IRB/IEC for review. The investigator must provide explanations and document any protocol deviations.

During the study, any changes to this study protocol must be submitted to the ethics committee. If necessary, corresponding changes should be simultaneously made to other study documents and submitted and/or be approved according to the pertinent requirements of the ethics committee. The investigator is responsible for submitting the interim reports regularly according to the pertinent requirements of the ethics committee. After the end of the trial, the completion should be informed to the ethics committee.

13.3 Confidentiality of Subject Information

During the course of this study, every effort shall be made to protect the privacy of all subjects. Study-related documents, study reports, publications, as well as any published data shall not include the names of subjects, except when required by law. To ensure the confidentiality of subjects' personal data, subject information shall be collected, transmitted, processed, and stored in accordance with applicable laws and regulations.

14. DROP-OUTS

All eligible subjects who have signed the informed consent form have the right to withdraw from the clinical trial at any time. Subjects who have not completed 1 cycle of study treatment and are unable to undergo safety and efficacy evaluations are considered drop-outs, regardless of the time and reason for withdrawal (withdrawals due to clear medical evidence of progression are not drop-outs). After a subject drops out, the investigator must document the reason for the drop-out in the eCRF, complete all the assessments possible, and document the last visit in the eCRF. Any drop-outs due to adverse reactions, which are determined to be related to the study drug during follow-up, must be documented in the eCRF and the investigator must be notified. Subjects who have undergone screening but withdrawn without obtaining a medication number are not considered drop-outs. When conducting the safety assessment, a statistical analysis should also be performed for subjects who complete an entire treatment cycle and have detailed documents available.

Subjects who withdraw are not permitted to be enrolled again, and the subjects' number may not be reused.

15. QUALITY CONTROL AND ASSURANCE

- The clinical study sites must be NMPA-approved drug research sites with clinical research qualifications;
- Study staff must be physicians trained for clinical trials, and must work under the supervision of senior professionals;
- Before the trial is started, the clinical wards must be inspected to ensure that standard requirements are met and first-aid equipment are available;
- Professional nursing staff are responsible for administering the drugs and they must be fully aware of the administration process to ensure subject compliance;
- Study sites must carry out the study in strict accordance with the protocol, and fill out the eCRF truthfully;
- The investigator should follow the standard operating procedure to ensure that all data are documented and reported accurately and completely, all eCRFs are entered correctly and consistent with source data, and the trial is implemented as per protocol;
- In the event of SAE, the CRA must inform all study sites immediately, and suspend the study whenever necessary;

16. PUBLICATION OF STUDY RESULTS

The study results belong to Jiangsu Province Hospital. Hengrui does not limit the publication of any collected or research information by investigators, regardless of whether the results are beneficial to the study drug or not.

17. TRIAL PROGRESS

Scheduled duration: Oct. 2017 to Apr. 2020.

18. TRIAL SITE AND TRIAL STAFF

Name:

Address:

Director of Clinical Trial Site:

Principal Investigator:

Study Staff: Chief Physician Tel.:

Associate Chief Physician Tel.:

Secretary of Clinical Trial Site: Tel.:

Appendix 1. Response Evaluation Criteria in Solid Tumors

Response Evaluation Criteria in Solid Tumors Version 1.1 (Excerpt)

(New Response Evaluation Criteria in Solid Tumors：Revised RECISTVersion 1.1)

Note: This appendix is translated internally and is for reference only. Please refer to the English version during practice.

1 BACKGROUND

Omitted

2 PURPOSE

Omitted

3 MEASURABILITY OF TUMOR AT BASELINE

3.1 Definition

At baseline, tumor lesions/lymph nodes will be categorized measurable or non-measurable as follows:

3.1.1 Measurable lesions

Tumor lesions: Must be accurately measured in at least one dimension (longest diameter is to be recorded) with a minimum size of:

- 10 mm by CT scan (CT scan slice thickness no greater than 5 mm)
- 10 mm caliper measurement by clinical exam (lesions which cannot be accurately measured with calipers should be recorded as non-measurable)
- 20 mm by chest X-ray
- Malignant lymph nodes: To be considered pathologically enlarged and measurable, a lymph node must be ≥ 15 mm in short axis when assessed by CT scan (CT scan slice thickness recommended to be no greater than 5 mm） At baseline and during follow-up, only the short axis will be measured and followed.

3.1.2 Non-measurable lesions

All other lesions, including small lesions (longest diameter < 10mm or pathological lymph nodes with ≥ 10 to < 15mm short axis) as well as truly non-measurable lesions. Non-measurable lesions include: meningeal disorder, ascites, pleural or pericardial effusion, breast carcinoma inflammatory, lymphangitis carcinomatosa of skin or lung, abdominal masses unable to be diagnosed or followed by imaging techniques, and cystic lesions.

3.1.3 Special considerations regarding lesion measurability

Bone lesions, cystic lesions, and lesions previously treated with local therapy require particular comment:

Bone lesions:

- Bone scan, PET scan or plain films are not considered adequate to measure bone lesions. However, these techniques can be used to confirm the presence or disappearance of bone lesions;
- Lytic lesions or mixed lytic-blastic lesions, with identifiable soft tissue components, that can be evaluated by tomography techniques such as CT or MRI can be considered as measurable lesions if the soft tissue component meets the definition of measurability described above;
- Blastic lesions are non-measurable.

Cystic lesions:

- Lesions that meet the criteria for radiographically defined simple cysts should not be considered as malignant lesions (neither measurable nor non-measurable) since they are, by definition, simple cysts.
- Cystic lesions thought to represent cystic metastases can be considered as measurable lesions, if they meet the definition of measurability described above. However, if noncystic lesions are present in the same patient, these are preferred for selection as target lesions.

Lesions with prior local treatment:

- Tumor lesions situated in a previously irradiated area, or in an area subjected to other locoregional therapy, are usually considered non-measurable unless there has been demonstrated progression in the lesion. Study protocols should detail the conditions under which such lesions would be considered measurable.

3.2 Methods of Measurement

3.2.1 Measurements of lesions

All measurements should be recorded in metric notation if clinically assessed. All baseline evaluations should be performed as close as possible to the treatment start and never more than 28 days (4 weeks) before the beginning of the treatment.

3.2.2 Method of evaluation

The same method and technique should be used to assess lesions at baseline and during follow-up. Imaging based evaluation should always be done rather than clinical examination unless the lesion(s) being followed cannot be imaged but are assessable by clinical exam.

Clinical lesions: Clinical lesions will only be considered measurable when they are superficial and ≥ 10mm diameter as assessed using calipers (e.g., nodule skin). For the case of cutaneous lesions, documentation by color photography including a ruler to estimate the size of the lesion is suggested. When lesions can be evaluated by both imaging and clinical examination, imaging evaluation should be undertaken since it is more objective and may also be reviewed at the end of the study.

Chest X-ray: Chest CT is preferred over chest X-ray, especially when tumor progression is an important clinical endpoint, since CT is more sensitive, particularly in identifying new lesions. Chest X-ray is only applicable when the measured lesion boundary is clear and the lungs are well ventilated.

CT and MRI: CT is currently the best available and reproducible method for efficacy evaluation. This guideline has defined measurability of lesions on CT scan based on the assumption that CT slice thickness is ≤ 5mm. When CT scans have slice thickness greater than 5 mm, the minimum size for a measurable lesion should be twice the slice thickness. MRI is also acceptable in certain situations (e.g., for whole body scans).

Ultrasound: Ultrasound should not be used as a method to measure lesion size. Ultrasound examinations are operation-dependent, and cannot be reproduced at a later date. It cannot be guaranteed that the same technique and measurements will be taken from one assessment to the next. If new lesions are identified by ultrasound in the course of the study, confirmation by CT or MRI is advised. If there is concern about radiation exposure at CT, MRI may be used instead.

Endoscopy, celioscopy: The utilization of these techniques for objective tumor evaluation is not advised. However, they can be useful to confirm CR when biopsies are obtained, or to determine relapse in trials where recurrence following CR or surgical excision is an endpoint.

Tumor markers: Tumor markers alone cannot be used to assess objective tumor response. However, if the marker levels exceed the upper normal limit at baseline, they must return to the normal levels for evaluation of complete response. Because tumor markers are disease specific, instructions for their measurement should be incorporated into protocols on a disease specific basis. Specific guidelines for both CA-125 response (in recurrent ovarian cancer) and PSA response (in recurrent prostate cancer), have been published. In addition, the Gynecologic Cancer Intergroup has developed CA-125 progression criteria which are to be integrated with objective tumor assessment for use in first-line trials in ovarian cancer.

Cytology/histology: These techniques can be used to differentiate between PR and CR in rare cases if required by protocol (for example, residual lesions in tumor types such as germ cell neoplasm, where known residual benign neoplasm can remain). When effusions are known to be a potential adverse effect of treatment (e.g., with certain taxane compounds or angiogenesis inhibitors), the cytological confirmation of the neoplastic origin of any effusion that appears or worsens during treatment can be considered if the measurable tumor has met the criteria for response or stable disease in order to differentiate between response (or stable disease) and progressive disease.

4 TUMOR RESPONSE ASSESSMENT

4.1 Assessment of Overall Tumor Load and Measurable Lesions

To assess objective response or future progression, it is necessary to estimate the overall tumor load at baseline and use this as a comparator for subsequent measurements. Only patients with measurable lesions at baseline should be included in protocols where objective response is the primary endpoint. Measurable lesion is defined by the presence of at least one measurable lesion. In trials where the primary endpoint is tumor progression (either time to progression or proportion with progression at a fixed date), the protocol must specify if enrollment is restricted to those with measurable lesions or whether patients with non-measurable lesions are also eligible.

4.2 Baseline Documentation of "Target" and "Non-target" Lesions

When more than one measurable lesion is present at baseline, all lesions representing all involved organs (≤ 5 in total, ≤ 2 per organ) should be identified as target lesions and will be recorded and measured at baseline (this means in instances where subjects have only 1 or 2 organ sites involved, a maximum of 2 and 4 lesions respectively will be recorded).

Target lesions should be selected on the basis of their size (lesions with the longest diameter), be representative of all involved organs, but in addition should be those that lend themselves to reproducible repeated measurements. It may be the case that, on occasion, the largest lesion does not lend itself to reproducible measurement in which circumstance the next largest lesion which can be measured reproducibly should be selected.

Lymph nodes merit special mention since they are normal tissues which may be visible by imaging even if not involved by tumor metastasis. Pathological nodes which are defined as measurable and may be identified as target lesions must meet the criterion of a short axis of ≥ 15 mm by CT scan. Only the short axis of these nodes needs to be measured at baseline. The short axis of the node is the diameter normally used by radiologists to judge if a node is involved by tumor metastasis. Nodule size is normally reported as two dimensions in the plane in which the image is obtained (for CT scan this is almost always the axial plane; for MRI the plane of acquisition may be axial, saggital or coronal). The smallest of these measures is the short axis. For example, an abdominal nodule which is reported as being 20mm x 30mm has a short axis of 20mm and qualifies as a malignant, measurable nodule. In this example, 20 mm should be recorded as the node measurement. Nodes with short axis ≥ 10 mm but < 15 mm) should be considered non-target lesions. Nodes that have a short axis < 10 mm are considered non-pathological and should not be recorded or followed.

A sum of the diameters (longest for non-nodal lesions, short axis for nodal lesions) for all target lesions will be calculated and reported as the baseline sum diameters. If lymph nodes are to be included in the sum, then as noted above, only the short axis is added into the sum. The baseline sum diameters will be used as reference.

All other lesions including pathological lymph nodes should be identified as non-target lesions, and while measurements are not required, they should be recorded at baseline. These lesions should be recorded as "present", "absent", or in rare cases "unequivocal progression". It is possible to record multiple nontarget lesions involving the same organ as a single item on the case record form (e.g., "multiple enlarged pelvic lymph nodes" or "multiple liver metastases").

4.3 Response Criteria

4.3.1 Evaluation of target lesions

Complete response (CR): Disappearance of all target lesions. Any pathological lymph nodes (whether target or non-target) must have reduction in short axis to < 10 mm.

Partial response (PR): At least a 30% decrease in the sum of diameters of target lesions, compared to baseline.

Progressive disease (PD): At least a 20% increase in the sum of diameters of target lesions, taking as reference the smallest sum on study (this includes the baseline sum if that is the smallest on study). In addition, the sum must also demonstrate an absolute increase of at least 5mm. (Note: the appearance of one or more new lesions is also considered progression).

Stable disease (SD): Neither sufficient shrinkage to qualify for PR nor sufficient increase to qualify for PD, taking as reference the smallest sum diameters while on study.

4.3.2 Precautions for target lesion assessment

Lymph nodes: Lymph nodes identified as target lesions should always have the actual short axis measurement recorded (measured in the same anatomical plane as the baseline examination), even if the nodes regress to below 10 mm on study. This means that when lymph nodules are included as target lesions, the sum of lesions may not be zero even if complete response criteria are met, since a normal lymph nodule is defined as having a short axis of < 10mm. Case report forms or other data collection methods may therefore be designed to have target nodal lesions recorded in a separate section where, in order to qualify for CR, each nodule must achieve a short axis of < 10mm. For PR, SD and PD, the actual short axis measurement of the nodules is to be included in the sum of target lesions.

Target lesions that become too small to measure: While on study, all lesions (nodal and non-nodal) recorded at baseline should have their actual measurements recorded at each subsequent evaluation, even when very small (e.g., 2mm). However, sometimes lesions or lymph nodes which are recorded as target lesions at baseline become so faint on CT scan that the radiologist may not feel comfortable assigning an exact measure and may report them as being "too small to measure". When this occurs, it is important that a value is recorded on the case report form. If it is the opinion of the radiologist that the lesion has likely disappeared, the measurement should be recorded as 0mm. If the lesion is believed to be present and is faintly seen but too small to measure, a default value of 5 mm could be assigned. (Note: It is less likely that this rule will be used for lymph nodes since they usually have a definable size when normal and are frequently surrounded by fat such as in the retroperitoneum; however, if a lymph node is believed to be present and is faintly seen but too small to measure, a default value of 5mm should be assigned in this circumstance as well). This default value is derived from the 5mm CT slice thickness (but should not be changed with varying CT slice thickness). The measurement of these lesions is potentially non-reproducible, therefore providing this default value will prevent false evaluation based upon measurement error. To reiterate, however, if the radiologist is able to provide an actual measure, that should be recorded, even if it is below 5mm.

Lesions that split or coalesce: When non-nodal lesions fragmented, the longest diameters of the fragmented portions should be added together to calculate the target lesion sum. Similarly, as lesions coalesce, a plane between them may be maintained that would aid in obtaining maximal diameter measurements of each individual lesion. If the lesions have truly coalesced such that they are no longer separable, the vector of the longest diameter in this instance should be the maximal longest diameter for the coalesced lesion.

4.3.3 Evaluation of non-target lesions

This section provides the definitions of the criteria used to determine the tumor response for the group of non-target lesions. While some non-target lesions may actually be measurable, they need not be measured and instead should be assessed only qualitatively at the time points specified in the protocol.

Complete response (CR): Disappearance of all non-target lesions and normalization of tumor marker level. All lymph nodes must be non-pathological in size (< 10 mm short axis).

Non-CR/Non-PD: Persistence of one or more non-target lesion(s) and/or maintenance of tumor marker level above the normal limits.

Progressive disease (PD): Unequivocal progression of existing non-target lesions. Note: the appearance of one or more new lesions is also considered disease progression.

4.3.4 Special notes on assessment of progression of non-target lesions

The concept of progression of non-target disease requires additional explanation as follows: When the patient also has measurable disease, to achieve unequivocal progression on the basis of the non-target disease, there must be an overall level of substantial worsening in non-target disease such that the overall tumor load has increased sufficiently to the point where treatment must be discontinued. A modest increase in the size of one or more non-target lesions is usually not sufficient to quality for unequivocal progression status. The designation of overall progression solely on the basis of change in non-target disease in the face of SD or PR of target disease will therefore be extremely rare.

When the patient has only non-measurable disease: This circumstance arises in some phase III trials when it is not a criterion of study inclusion to have measurable disease. The same general concepts apply here as noted above, however, in this instance there is no measurable disease assessment. Because worsening in non-target disease cannot be easily quantified (by definition: if all lesions are truly non-measurable), a useful test that can be applied when assessing patients for unequivocal progression is to consider if the increase in overall disease load based on the change in non-measurable disease is comparable in magnitude to the increase that would be required to declare PD for measurable disease. For example, an increase in tumor load representing an additional 73% increase in volume (which is equivalent to a 20% increase diameter in a measurable lesion). Examples include an increase in a peritoneal effusion from trace to large, an increase in lymphangiopathy from localized to widespread, or may be described in protocols as "sufficient to require a change in treatment". Examples include an increase in a pleural effusion from trace to large, an increase in lymphangitic disease from localized to widespread, or may be described in protocols as "sufficient to require a change in therapy". If unequivocal progression is seen, the patient should be considered to have had overall PD at that point. While it would be ideal to have objective criteria to apply to non-measurable disease, the very nature of that disease makes it impossible to do so, therefore the increase must be substantial.

4.3.5 New Lesions

The appearance of new malignant lesions denotes disease progression; therefore, some comments on detection of new lesions are important. There are no specific criteria for the identification of radiographically detected lesions; however, the finding of a new lesion should be unequivocal. For example, it should not be attributable to differences in scanning technique, change in imaging modality, or findings thought to represent something other than tumor (for example, some new bone lesions that may be simply healing, or re-occurrence of pre-existing lesions). This is particularly important when the patient's baseline lesions show partial or complete response. For example, necrosis of a liver lesion may be reported on a CT scan report as a new cystic lesion, which it is not.

A lesion identified on a follow-up study that was not scanned at baseline is considered a new lesion and will indicate disease progression. An example of this is the patient who has visceral disease at baseline and while on study has a CT or MRI brain ordered which reveals metastases. The patient's brain metastases are considered to be evidence of PD even if he/she did not have brain imaging at baseline.

If a new lesion is equivocal, for example because of its small size, continued treatment and follow-up evaluation are required to clarify if it represents a truly new disease. If repeated scans confirm there is definitely a new lesion, then progression should be declared using the date of the initial identification.

While FDG-PET response assessments generally need additional study, it is sometimes reasonable to incorporate the use of FDG-PET scanning to complement CT scanning in assessment of progression (particularly possible new disease). New lesions on the basis of FDG-PET imaging can be identified according to the following process:

Negative FDG-PET at baseline, with a positive FDG-PET at follow-up is a sign of PD based on a new lesion.

No FDG-PET at baseline and a positive FDG-PET at follow-up:

If the positive FDG-PET at follow-up corresponds to a new site of disease confirmed by CT, PD is confirmed.

If the positive FDG-PET at follow-up is not confirmed as a new site of disease on CT, additional follow-up CT scans are needed to determine if there is truly progression occurring at that site (if so, the date of PD will be the date of the initial abnormal FDG-PET scan).

If the positive FDG-PET at follow-up corresponds to a pre-existing site of disease on CT that is not progressing on the basis of the imaging examination, this is not PD.

4.4 Evaluation of Best Overall Response

The best overall response is the best response recorded from the start of the trial until the end of trial taking into account any necessary requirement for confirmation. On occasion a response may not be documented until after the end of treatment so protocols should be clear if post-treatment assessments are to be considered in the evaluation of best overall response. Protocols must specify how any new treatment introduced before progression will affect best response evaluation. The patient's best overall response evaluation will depend on the findings of both target and non-target diseases and will also take into consideration the characteristics of new lesions. Furthermore, depending on the nature of the study and the protocol requirements, it may also require confirmatory measurement. Specifically, in non-randomized trials where response is the primary endpoint, confirmation of PR or CR is needed to determine either one is the best overall response.

4.4.1 Time point response

It is assumed that at each time point specified in protocol, an efficacy response occurs. Table 1 provides a summary of the overall response status calculation at each time point for patients who have measurable disease at baseline.

Table 1. Time point response: patients with target (+/– non-target) disease.

Target Lesions	Non-Target Lesions	New Lesions	Overall Response
CR	CR	No	CR
CR	Non-CR/Non-PD	No	PR
CR	Not evaluable	No	PR
PR	Non-PD or not all evaluable	No	PR
SD	Non-PD or not all evaluable	No	SD
Not all evaluable	Non-PD	No	NE
PD	Any	Yes or No	PD
Any	PD	Yes or No	PD
Any	Any	Yes	PD

CR = complete response, PR = partial response, SD = stable disease, PD = progressive disease, and NE = not evaluable.

If patient does not have measurable lesions (no target lesions), refer to Table 2.

Table 2. Time point response: patients with non-target disease only.

Non-target Lesions	New Lesions	Overall Response
CR	No	CR
Non-CR/Non-PD	No	Non-CR/Non-PD[a]
Not all evaluable	No	Not evaluable
Equivocal PD	Yes or No	PD
Any	Yes	PD

[a]: "Non-CR/non-PD" is preferred over SD for non-target disease. Since SD is increasingly used as an endpoint for efficacy evaluation, non-CR/non-PD response is developed to address the absence of lesion measurability.

4.4.2 Missing assessments and inevaluable designation

When no imaging/measurement is done at all at a particular time point, the patient is not evaluable at that time point. If only a subset of lesion measurements is made at an evaluation, usually the case is also considered not evaluable at that time point, unless a convincing argument can be made that the contribution of the individual missing lesion(s) has/have no effect on the assigned time point response. This would be most likely to happen in the case of PD. For example, if a patient had a baseline sum of 50 mm with three measured lesions and only two lesions were assessed at subsequent follow-up, but those gave a sum of 80 mm, the patient will have achieved PD status, regardless of the contribution of the missing lesion.

4.4.3 Best overall response: all time points

The best overall response is determined once all the data for the patient are known.

Best overall response determination in trials where confirmation of complete or partial response is not required: Best response in these trials is defined as the best response across all time points (for example, a patient who has SD in evaluation at Cycle 1, PR at Cycle 2, and PD at the last cycle has a best overall response of PR). When SD is believed to be best response, it must also meet the protocol specified minimum time calculated from baseline. If the minimum time is not met when SD is otherwise the best overall response, the patient's best overall response depends on the subsequent assessments. For example, a patient who has SD at Cycle 1, PD at Cycle 2 and does not meet minimum duration for SD, will have a best overall response of PD. The same patient lost to follow-up after the first SD assessment would be considered not evaluable.

Best overall response determination in trials where confirmation of complete or partial response is required: Complete or partial responses may be claimed only if the criteria for each are met at a subsequent time point as specified in the protocol (generally 4 weeks later). In this circumstance, the best overall response can be interpreted as in Table 3.

Chen X, *et al. J Immunother Cancer* 2022; 8:e001240. doi: 10.1136/jitc-2020-001240

Table 3. Best overall response when confirmation of CR and PR required.

Overall Response at First Time Point	Overall Response at Subsequent Time Point	Best Overall Response
CR	CR	CR
CR	PR	SD, PD or PR[a]
CR	SD	SD (provided minimum criteria for SD duration met, otherwise, PD)
CR	PD	SD (provided minimum criteria for SD duration met, otherwise, PD)
CR	NE	SD (provided minimum criteria for SD duration met, otherwise, NE)
PR	CR	PR
PR	PR	PR
PR	SD	SD
PR	PD	SD (provided minimum criteria for SD duration met, otherwise, PD)
PR	NE	SD (provided minimum criteria for SD duration met, otherwise, NE)
NE	NE	NE

CR = complete response, PR = partial response, SD = stable disease, PD = progressive disease, and NE = not evaluable.
[a]: If a CR is truly met at first time point, then any disease seen at a subsequent time point, even disease meeting PR criteria relative to baseline, makes the disease PD at that point (since disease must have reappeared after CR). Best response would depend on whether minimum duration for SD is met. However, sometimes CR may be claimed when subsequent scans suggest small lesions were likely still present and in fact the patient had PR, not CR at the first time point. Under these circumstances, the original CR should be changed to PR and the best response is PR.

4.4.4 Special notes on response assessment

When nodal disease is included in the sum of target lesions and the nodes decrease to "normal" size (< 10 mm), they may still have a measurement reported on scans. This measurement should be recorded even though the nodules are normal in order not to overstate progression should it be based on increase in size of the nodes. As noted earlier, this means that patients with CR may not have "zero" recorded on the case report form (CRF).

In trials where confirmation of response is required, repeated "NE" time point evaluations may complicate best response determination. The analysis plan for the trial must address how missing data/evaluations will be addressed in determination of response and progression. For example, in most trials it is reasonable to consider a patient with time point responses of PR-NE-PR as a confirmed response.

Chen X, *et al. J Immunother Cancer* 2022; 8:e001240. doi: 10.1136/jitc-2020-001240

Patients with an overall deterioration of health status requiring discontinuation of treatment without objective evidence of disease progression at that time should be reported as symptomatic deterioration. Efforts should be made to evaluate objective progression even after discontinuation of treatment. Symptomatic deterioration is not a description of an objective response: it is a reason for discontinuation of treatment. The objective response status of such patients is to be determined by evaluation of target and non-target disease as shown in Tables 1-3.

Conditions that are defined as early progression, early death and not evaluable are study specific and should be clearly described in each protocol (depending on treatment duration and treatment cycle).

In some circumstances it may be difficult to distinguish residual lesions from normal tissues. When the evaluation of complete response depends upon this definition, it is recommended that the local lesion be investigated before assigning a status of complete response. FDG-PET may be used to confirm a response to a CR in a manner similar to a biopsy in cases where a residual radiographic abnormality is thought to represent fibrosis or scarring. The use of FDG-PET in this circumstance should be prospectively described in the protocol and supported by disease specific medical literature for the indication. However, it must be acknowledged that both approaches may lead to false positive CR due to limitations of FDG-PET and biopsy resolution/sensitivity.

For equivocal findings of progression (e.g., very small and uncertain new lesions; cystic changes or necrosis in existing lesions), treatment may continue until the next scheduled evaluation. If at the next scheduled evaluation, progression is confirmed, the date of progression should be the earlier date when progression was suspected.

4.5 Frequency of Tumor Re-evaluation

Frequency of tumor re-evaluation during treatment should be protocol-specific and consistent with the type and schedule of treatment. However, in the Phase II trials where the beneficial effect of treatment is not known, follow-ups for every 6-8 weeks (timed to coincide with the end of a cycle) is reasonable. Interval adjustments could be justified in specific regimens or circumstances. The protocol should specify which organ sites are to be evaluated at baseline (usually those most likely to be involved with metastatic disease for the tumor type under study) and how often evaluations are repeated. Normally, all target and non-target sites are evaluated at each assessment. In selected circumstances, certain non-target organs may be evaluated less frequently. For example, bone scans may need to be repeated only when CR is identified in target disease or when progression in bone is suspected.

Chen X, *et al. J Immunother Cancer* 2022; 8:e001240. doi: 10.1136/jitc-2020-001240

After treatment, the need for tumor re-evaluations depends on whether the trial has as made the response rate or the time to an event (progression/death) an endpoint. If "time to an event" (e.g., TTP/DFS/PFS) is the main endpoint of the study, then routine scheduled re-evaluation of protocol specified sites of disease is warranted. In randomized comparative trials in particular, the scheduled assessments should be performed as identified on a calendar schedule (for example: every 6-8 weeks on treatment or every 3-4 months after treatment) and should not be affected by delays in therapy, drug holidays or any other events that might lead to imbalance in a treatment group in the timing of disease assessment.

4.6 Confirmatory Measurement/Duration of Response

4.6.1 Confirmation

In non-randomized trials where response is the primary endpoint, confirmation of PR and CR is required to ensure responses identified are not the result of measurement error. This will also permit appropriate interpretation of results in the context of historical data where response has traditionally required confirmation in such trials. However, in all other circumstances, i.e., in randomized trials (phase II or III) or studies where stable disease or progression are the primary endpoints, confirmation of response is not required since it will not add value to the interpretation of trial results. However, elimination of the requirement for response confirmation may increase the importance of central review to protect against bias, in particular in studies which are not blinded.

In the case of SD, measurements must have met the SD criteria at least once after study entry at a minimum interval (in general not less than 6–8 weeks) that is defined in the study protocol.

4.6.2 Duration of overall response

The duration of overall response is measured from the time measurement criteria are first met for CR/PR (whichever is first recorded) until the first date that recurrent or progressive disease is objectively documented (taking as reference for progressive disease the smallest measurements recorded on study). The duration of overall complete response is measured from the time criteria are first met for CR until the first date that recurrent or progressive disease is truly documented.

4.6.3 Duration of stable disease

Stable disease is measured from the start of the treatment (in randomized trials, from date of randomization) until the criteria for progression are met, taking as reference the smallest sum on study (if the baseline sum is the smallest, this is the reference for calculation of PD). The clinical relevance of the duration of stable disease varies in different studies and diseases. If the proportion of patients achieving stable disease for a minimum period of time is an endpoint in a particular trial, the protocol should specify the minimal time interval required between two measurements for determination of stable disease.

Note: The duration of response and stable disease as well as the progression-free survival are influenced by the frequency of follow-up after baseline evaluation. It is not in the scope of this guideline to define a standard follow-up frequency. The frequency should take into account many parameters including disease types and stages, treatment cycle and standard practice. However, these limitations of the precision of the measured endpoint should be taken into account if comparisons between trials are to be made.

4.7 PFS/TTP

4.7.1 Phase II trials

This guideline is focused primarily on the use of objective response as study endpoints for phase II trials. In some circumstances, response rate may not be the optimal method to assess the potential anticancer activity of new agents/regimens. In such cases, PFS/PPF at landmark time points might be considered appropriate alternatives to provide an initial signal of biologic effect of new agents. It is clear, however, that in an uncontrolled trial, these measures are subject to criticism since an apparently promising observation may be related to biological factors such as patient selection and not the impact of the intervention. Thus, phase II screening trials utilizing these endpoints are best designed with a randomized control. Exceptions may exist where the behavior patterns of certain cancers are so consistent (and usually consistently poor), that a non-randomized trial is justifiable. However, in these cases, it will be essential to document with care the basis for estimating the expected PFS or PPF in the absence of a treatment effect.

Appendix 2. Child-Pugh Score

Indicator	Score		
	1	2	3
Hepatic encephalopathy	☐No	☐Grade 1-2	☐Grade 3-4
Ascites	☐No	☐Mild	☐Moderate and above
Prothrombin time prolonged or INR	☐1-3 seconds<br>☐＜1.7	☐4-6 seconds<br>☐1.7-2.3	☐> 6 seconds<br>☐＞2.3
Bilirubin total (μmol/L)	☐＜34	☐34-51	☐＞51
Serum albumin (g/L)	☐＞35	☐28-35	☐＜28

Appendix 3. Performance Status (ECOG)

(Eastern Cooperative Oncology Group)

Score	Description
0	Asymptomatic, fully active, able to carry on all performance without restriction.
1	Symptomatic, restricted in physically strenuous activity but ambulatory and able to carry out work of a light or sedentary nature, e.g., light house work, office work.
2	Symptomatic, ambulatory and capable of all self-care but unable to carry out any physical activities; up and about more than 50% of waking hours (confined to bed < 50% of waking hours).
3	Symptomatic, capable of only limited self-care; confined to bed or chair more than 50% of waking hours, but not totally confined to bed.
4	Completely disabled; cannot carry on any self-care; totally confined to bed or chair.
5	Death.

Chen X, *et al. J Immunother Cancer* 2022; 8:e001240. doi: 10.1136/jitc-2020-001240

Appendix 4. New York Heart Association (NYHA) Functional Classification: Cardiac function is categorized into four classes, and heart failure is categorized into three degrees.

**Heart Function Classification**	**Judgment**			**Grade of Heart Failure (cardiac insufficiency)**
	**Cardiac Reserve**	**Physical Activity (symptoms)**	**Signs of Heart Disease and Cardiac Insufficiency**	
Class I	Normal	No limitation in physical activity. Ordinary physical activity does not cause undue fatigue, asthenia, palpitation, dyspnea or anginal pain.	No signs of heart failure.	Cardiac functional compensatory period
Class II	Mild decrease	Slight limitation of physical activity, but comfortable at rest. Ordinary physical activity (such as walking at normal speed for 500-1000 m or climbing 3-4 floors) results in fatigue, palpitation, dyspnea, or anginal pain, which resolve after rest.	Signs of heart failure, such as increase in heart rate and mild hepatomegaly.	Grade I (mild)
Class III	Moderate decrease	Marked limitation of physical activity. Comfortable at rest. Less than ordinary activity (such as usual housework, walking at normal speed for 500-1000 m or climbing 2 floors) results in palpitation, dyspnea, or anginal pain, which alleviate after bed rest but do not completely disappear.	Signs of heart failure, such as hepatomegaly and edema	Grade II (moderate)
Class IV	Severe decrease	Unable to carry on any physical activity. Symptoms of fatigue, palpitation, dyspnea, or anginal pain may be present at rest.	Significant signs of heart failure	Grade III (severe)
