# Supplementary material for: Camrelizumab plus gemcitabine and oxaliplatin (GEMOX) in patients with advanced biliary tract cancer: a single-arm, open-label, phase II trial
Source: J Immunother Cancer. 2020 Nov 10;8(2):e001240. doi: 10.1136/jitc-2020-001240 (PMC7656907; doi:10.1136/jitc-2020-001240)
Supplement: Supplementary data [file jitc-2020-001240supp001.pdf]

# Supplementary appendix

Chen XF, Wu XF, Wu H, et al. Camrelizumab plus gemcitabine and oxaliplatin (GEMOX) in patients with advanced biliary tract cancer: a single-arm open-label, phase 2 trial.

**Supplementary Table S1.** Patient baseline characteristics.

**Supplementary Table S2.** Responses and survival by PD-L1 expression and tumor mutational burden.

**Supplementary Figure S1.** Kaplan-Meier Estimates of Progression-Free Survival and Overall Survival by PD-L1 Expression Status.

**Supplementary Figure S2.** Immune cells in center or invasive margin between responders and non-responders.

**Supplementary Figure S3.** Kaplan-Meier Estimates of Progression-Free Survival and Overall Survival by TMB Status or gene mutation.

**Supplementary Figure S4.** The association between survival and ctDNA status or bTMB.

**Supplementary Figure S5.** The association between survival and ctDNA status in non-responders and responders.

This supplementary material has been provided by the authors to give readers additional information about their work.

| **Characteristic** | **camrelizumab + GEMOX (N = 37)** |
|---|---|
| Age – yr | |
| Median | 64 |
| Range | 41-74 |
| Primary site – no. (%) | |
| Cholangiocarcinoma | 22 (59) |
| Gallbladder cancer | 15 (41) |
| Gender – no. (%) | |
| Male | 26 (70) |
| Female | 11 (30) |
| Disease stage – no. (%) | |
| IV | 37(100) |
| Sites of distant metastases | |
| Liver | 34 (92) |
| Lymph node | 13 (35) |
| Peritoneum | 12 (32) |
| Lung | 6 (16) |
| ECOG performance-status score – no. (%) | |
| 0 | 13 (35) |
| 1 | 24 (65) |

**Supplementary Table S1. Patient baseline characteristics**

| | Overall responses | Median progression-free survival | Median overall survival |
|---|---|---|---|
| **Tumor proportion score** | | | |
| <1% | 14/26 (53.8) | 6.0 (4.2-7.1) | 11.9 (8.4-21.9) |
| ≥1% | 4/5 (80) | 9.0 (8.5-NR) | 17.8 (14.2-NR) |
| Total | 18/31 (58.1) | 6.1 (4.6-8.5) | 13.0 (9.1-21.0) |
| **Immune proportion score** | | | |
| <1% | 13/23 (56.5) | 6.0 (4.2-7.1) | 11.1 (8.4-21.0) |
| ≥1% | 5/8 (62.5) | 8.7 (4.6-NR) | 19.9 (14.2-NR) |
| Total | 18/31 (58.1) | 6.1 (4.6-8.5) | 13.0 (9.1-21.0) |
| **Tumor mutational burden** | | | |
| Bottom 75% | 12/24 (50) | 6.0 (4.3-9.0) | 11.4 (8.4-21.0) |
| Top 25% | 6/10 (60) | 6.1 (4.0-NR) | 13.1 (8.5-NR) |
| Total | 18/34 (53) | 6.0 (4.6-8.4) | 12.0 (9.1-17.8) |
| **Blood mutational burden** | | | |
| Bottom 75% | 13/22 (59.1) | 6.4 (5.4-9.1) | 10.8 (7.6-19.5) |
| Top 25% | 4/8 (50) | 4.1 (2.4-NR) | 11.6 (8.5-NR) |
| Total | 17/30 (56.7) | 6.1 (4.3-8.4) | 10.8 (8.4-14.2) |
| **Circulating tumor DNA** | | | |
| Positive at 8 weeks | 7/17 (41.2) | 4.3 (3.9-6.2) | 9.1 (7.6-NR) |
| Negative at 8 weeks | 10/13 (76.9) | 7.3 (6.2-NR) | 13.0 (8.5-NR) |
| Total | 17/30 (56.7) | 5.7 (4.3-7.3) | 10.8 (8.0-14.2) |

Data consists of number of overall responses/number of patients (%). NR, not reached.

**Supplementary Table S2. Responses and survival by PD-L1 expression and tumor mutational burden**

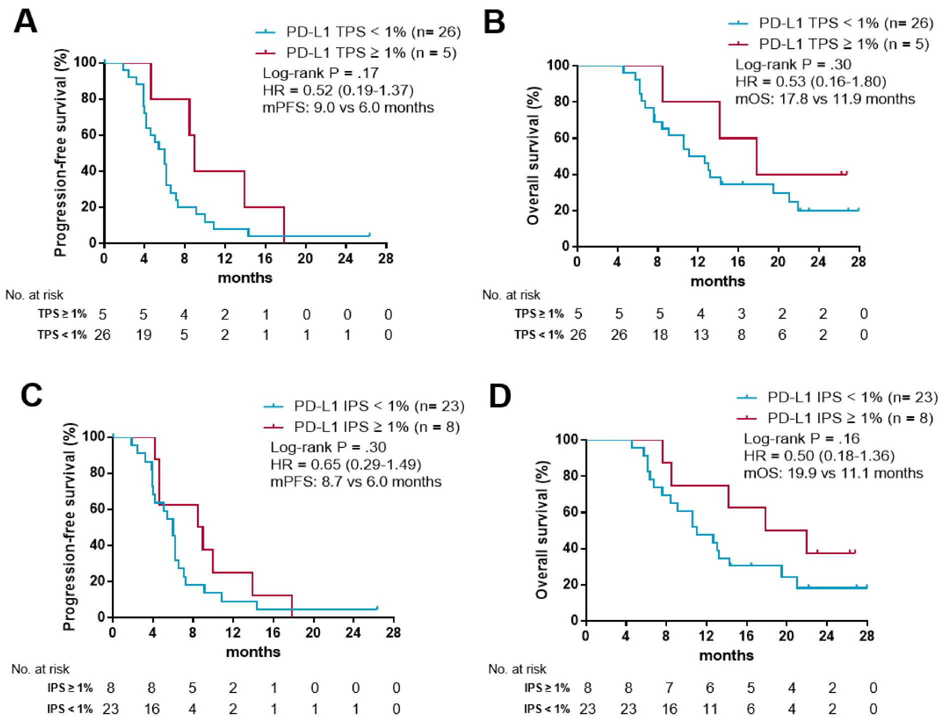


**Supplementary Figure S1. Kaplan-Meier Estimates of Progression-Free Survival and Overall Survival by PD-L1 Expression Status.** (A) Kaplan-Meier survival curves of PFS comparing patients treated with camrelizumab plus GEMOX with PD-L1 tumor proportion scores of at least 1% and less than 1%. (B) Kaplan-Meier survival curves of OS comparing patients treated with camrelizumab plus GEMOX with PD-L1 tumor proportion scores of at least 1% and less than 1%. (C) Kaplan-Meier survival curves of PFS comparing patients treated with camrelizumab plus GEMOX with PD-L1 immune proportion scores of at least 1% and less than 1%. (D) Kaplan-Meier survival curves of OS comparing patients treated with camrelizumab plus GEMOX with PD-L1 immune proportion scores of at least 1% and less than 1%.

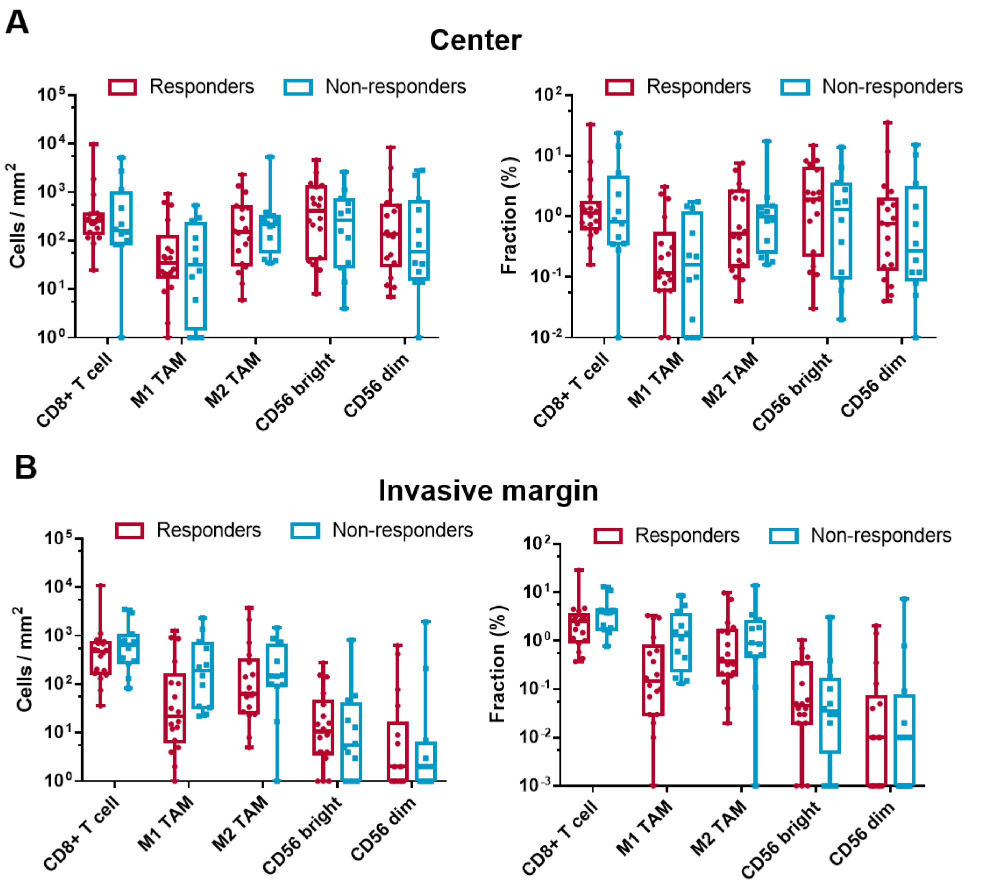


**Supplementary Figure S2. Immune cells in center or invasive margin between responders and non-responders.** (A) Comparison of immune cells in tumor center between responders and non-responders. (B) Comparisons of immune cells in invasive margin between responders and non-responders.

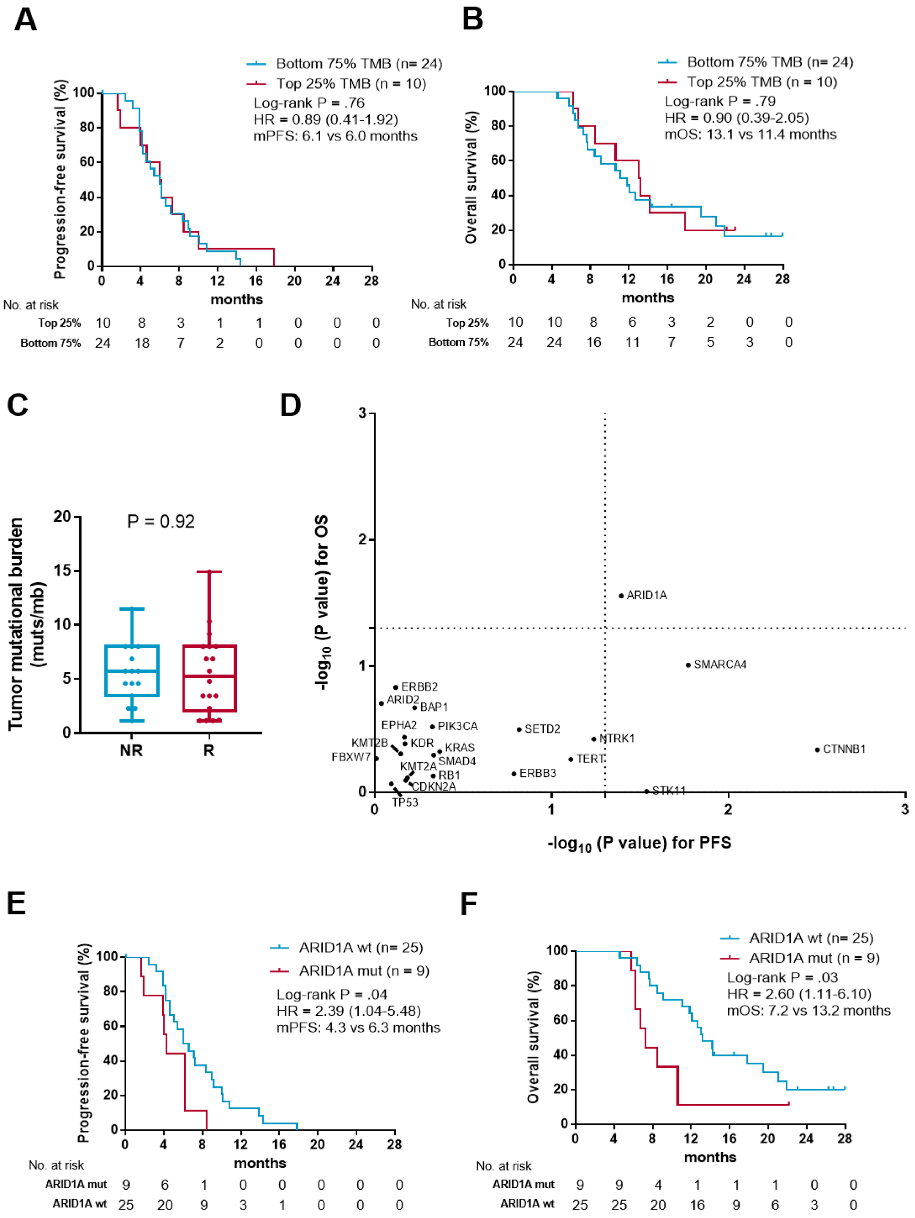


**Supplementary Figure S3. Kaplan-Meier Estimates of Progression-Free Survival and Overall Survival by TMB Status or gene mutation.** (A) Kaplan-Meier survival curves of PFS comparing patients treated with camrelizumab plus GEMOX with top 25% TMB and bottom 75% TMB. (B) Kaplan-Meier survival curves of OS comparing patients treated with camrelizumab plus GEMOX with top 25% TMB and bottom 75% TMB. (C) Comparison of TMB in responders and non-responders. (D) Comparisons of the association between individual altered genes and PFS or OS. (E)

Kaplan-Meier survival curves of PFS comparing patients treated with camrelizumab plus GEMOX with or without ARID1A mutation. (F) Kaplan-Meier survival curves of OS comparing patients treated with camrelizumab plus GEMOX with or without ARID1A mutation.

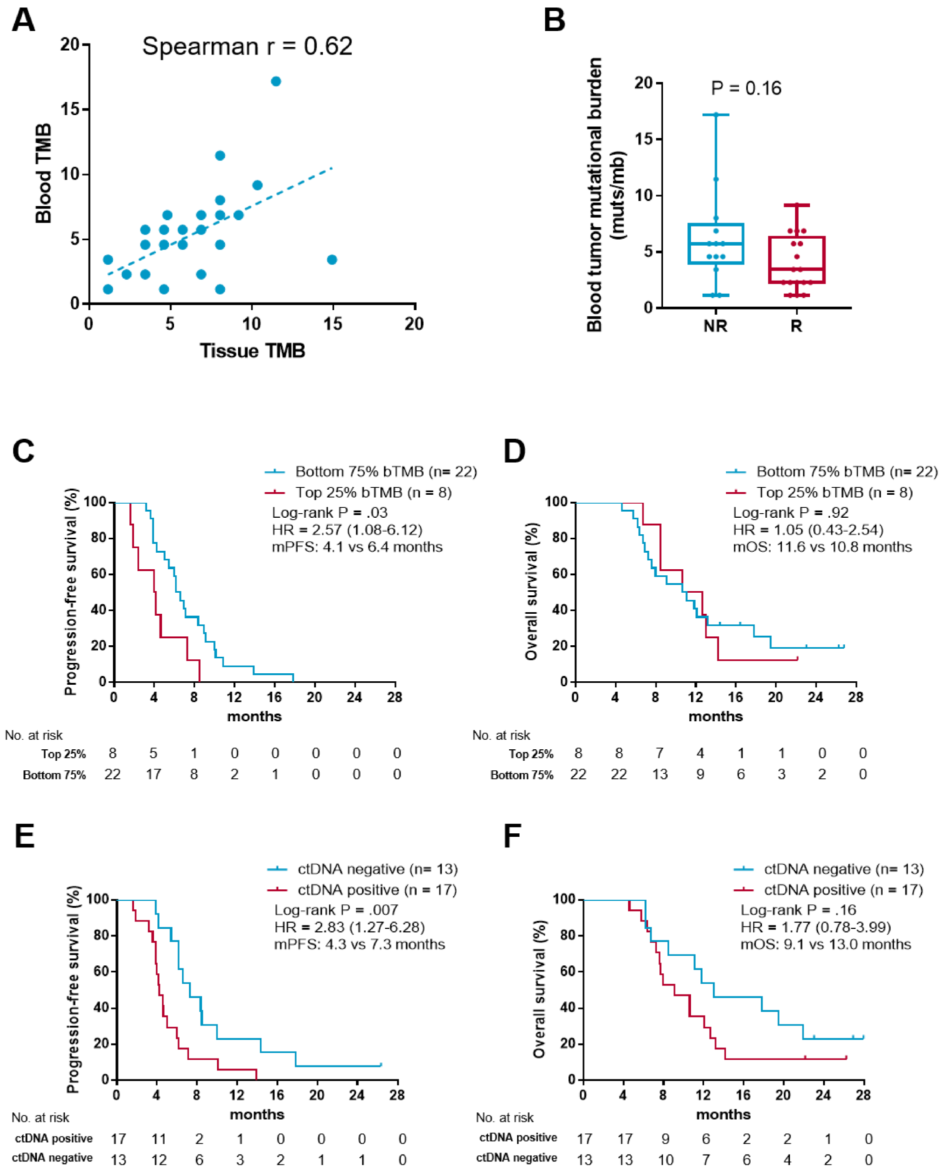


**Supplementary Figure S4. The association between survival and ctDNA statue or bTMB.** (A) The correlation between blood TMB and tissue TMB. (B) Comparison of bTMB between responders and non-responders. (C) Kaplan-Meier survival curves of PFS comparing patients treated with camrelizumab plus GEMOX with top 25% TMB and bottom 75% bTMB. (D) Kaplan-Meier survival curves of OS comparing patients treated with camrelizumab plus GEMOX with top 25% TMB and bottom 75% bTMB. (E) Kaplan-Meier survival curves of PFS comparing patients with positive post-treatment ctDNA and negative post-treatment ctDNA. (F) Kaplan-Meier survival

curves of OS comparing patients with positive post-treatment ctDNA and negative post-treatment ctDNA.

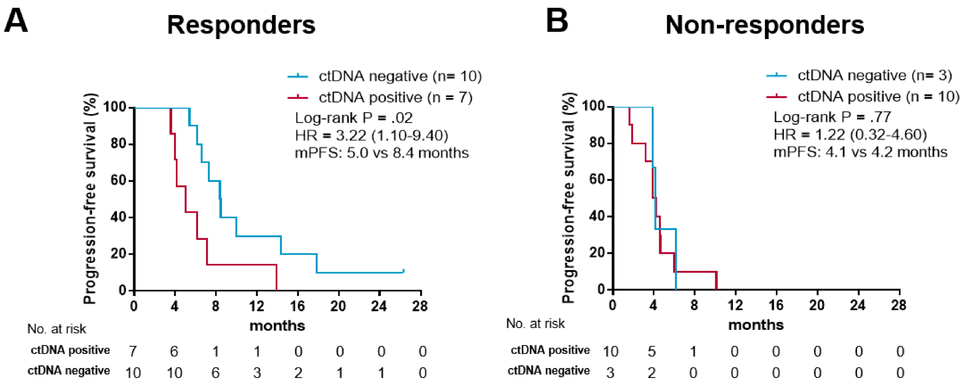


**Supplementary Figure S5.** The association between survival and ctDNA status in non-responders and responders. (A) Kaplan-Meier survival curves of PFS comparing patients with positive post-treatment ctDNA and negative post-treatment ctDNA in responders. (B) Kaplan-Meier survival curves of PFS comparing patients with positive post-treatment ctDNA and negative post-treatment ctDNA in non-responders.
